# Pre- and Post-harvest Factors Affecting Glucosinolate Content in Broccoli

**DOI:** 10.3389/fnut.2020.00147

**Published:** 2020-09-10

**Authors:** Riadh Ilahy, Imen Tlili, Zoltán Pék, Anna Montefusco, Mohammed Wasim Siddiqui, Fozia Homa, Chafik Hdider, Thouraya R'Him, Helyes Lajos, Marcello Salvatore Lenucci

**Affiliations:** ^1^Laboratory of Horticulture, National Agricultural Research Institute of Tunisia (INRAT), University of Carthage, Tunis, Tunisia; ^2^Laboratory of Horticulture, Faculty of Agricultural and Environmental Sciences, Horticultural Institute, Szent István University, Budapest, Hungary; ^3^Dipartimento di Scienze e Tecnologie Biologiche ed Ambientali, Università del Salento (DiSTeBA), Lecce, Italy; ^4^Department of Food Science and Postharvest Technology, Bihar Agricultural University, Bhagalpur, India; ^5^Department of Statistics, Mathematics, and Computer Application, Bihar Agricultural University, Bhagalpur, India

**Keywords:** aliphatic, indole and aromatic glucosinolates, biosynthetic pathways, broccoli, *Brassica oleracea* L var. *italica*, pre-harvest and post-harvest management

## Abstract

Owing to several presumed health-promoting biological activities, increased attention is being given to natural plant chemicals, especially those frequently entering the human diet. Glucosinolates (GLs) are the main bioactive compounds found in broccoli (*Brassica oleracea* L. var. *italica* Plenck). Their regular dietary assumption has been correlated with reduced risk of various types of neoplasms (lung, colon, pancreatic, breast, bladder, and prostate cancers), some degenerative diseases, such as Alzheimer's, and decreased incidence of cardiovascular pathologies. GL's synthesis pathway and regulation mechanism have been elucidated mainly in *Arabidopsis*. However, nearly 56 putative genes have been identified as involved in the *B*. *oleracea* GL pathway. It is widely recognized that there are several pre-harvest (genotype, growing environment, cultural practices, ripening stage, etc.) and post-harvest (harvesting, post-harvest treatments, packaging, storage, etc.) factors that affect GL synthesis, profiles, and levels in broccoli. Understanding how these factors act and interact in driving GL accumulation in the edible parts is essential for developing new broccoli cultivars with improved health-promoting bioactivity. In this regard, any systematic and comprehensive review outlining the effects of pre- and post-harvest factors on the accumulation of GLs in broccoli is not yet available. Thus, the goal of this paper is to fill this gap by giving a synoptic overview of the most relevant and recent literature. The existence of substantial cultivar-to-cultivar variation in GL content in response to pre-harvest factors and post-harvest manipulations has been highlighted and discussed. The paper also stresses the need for adapting particular pre- and post-harvest procedures for each particular genotype in order to maintain nutritious, fresh-like quality throughout the broccoli value chain.

## General Overview and Commercial Importance of Broccoli

The *Brassicaceae* family comprises 52 tribes, 351 genera, and 3,977 species, some of great economic importance (BrassiBase, https://brassibase.cos.uni-heidelberg.de). Cultivated varieties of *Brassica oleracea* L. share a common genome comprising nine chromosomes and present an outstanding diversification of morphotypes, including broccoli (var. *italica* Plenk), Brussels sprouts [var. *gemmifera* (DC.) Zenker], cabbage (var. *capitata* L.), cauliflower (var. *botrytis* L.), kale (var. *medullosa* Thell.), kohlrabi (var. *gongylodes* L.), and several endemisms. All these were likely shaped through a process of human domestication starting from a simple leafy type ancestor (kale, var. *viridis* L.), resulting in arrested development, enlargement, and morphological alteration of the epigeous organs (inflorescences, leaves, stems, or buds) (1, 2).

**Figure 1:**
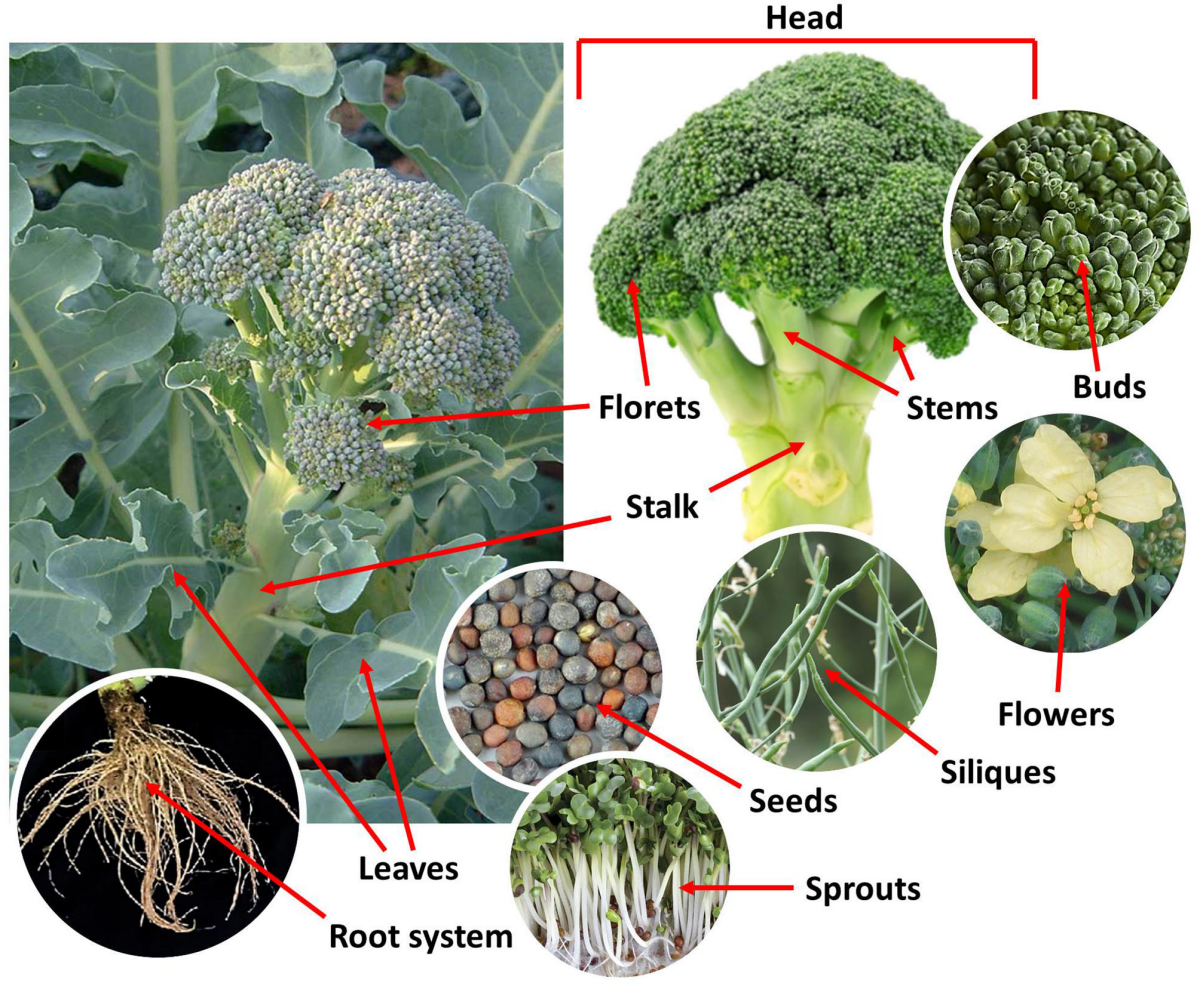
Anatomy of broccoli (*Brassica oleracea* L., var. *Italica*).

Broccoli is a annual, cool season, herbaceous crop with optimum growth temperatures between 13 and 20°C (3). It grows 60–90 cm tall and forms an upright and branching thick green stalk with leathery, oblong gray-blue to green rosette basal leaves. At the end of the central axis and the branches (stems), broccoli has dense green edible clusters of flower buds (florets) that, if left unharvested, bloom in yellow flowers with four petals and produce silique fruits. The florets and the upper stems form the inflorescence (head) of the broccoli, which constitutes the commonly consumed organ (Figure 1). The word broccoli is derived from the Latin word “*brachium*” (brocco in modern Italian), which means branch and refers to the frequent sprouts making up the head (4).

Although broccoli domestication dates back to the ancient Italian Etruscan civilization, its large-scale commercial cultivation started about a century ago in California and spread widely all over the world after World War II, adapting to local climatic conditions and environments (4–6).

**Figure 2:**
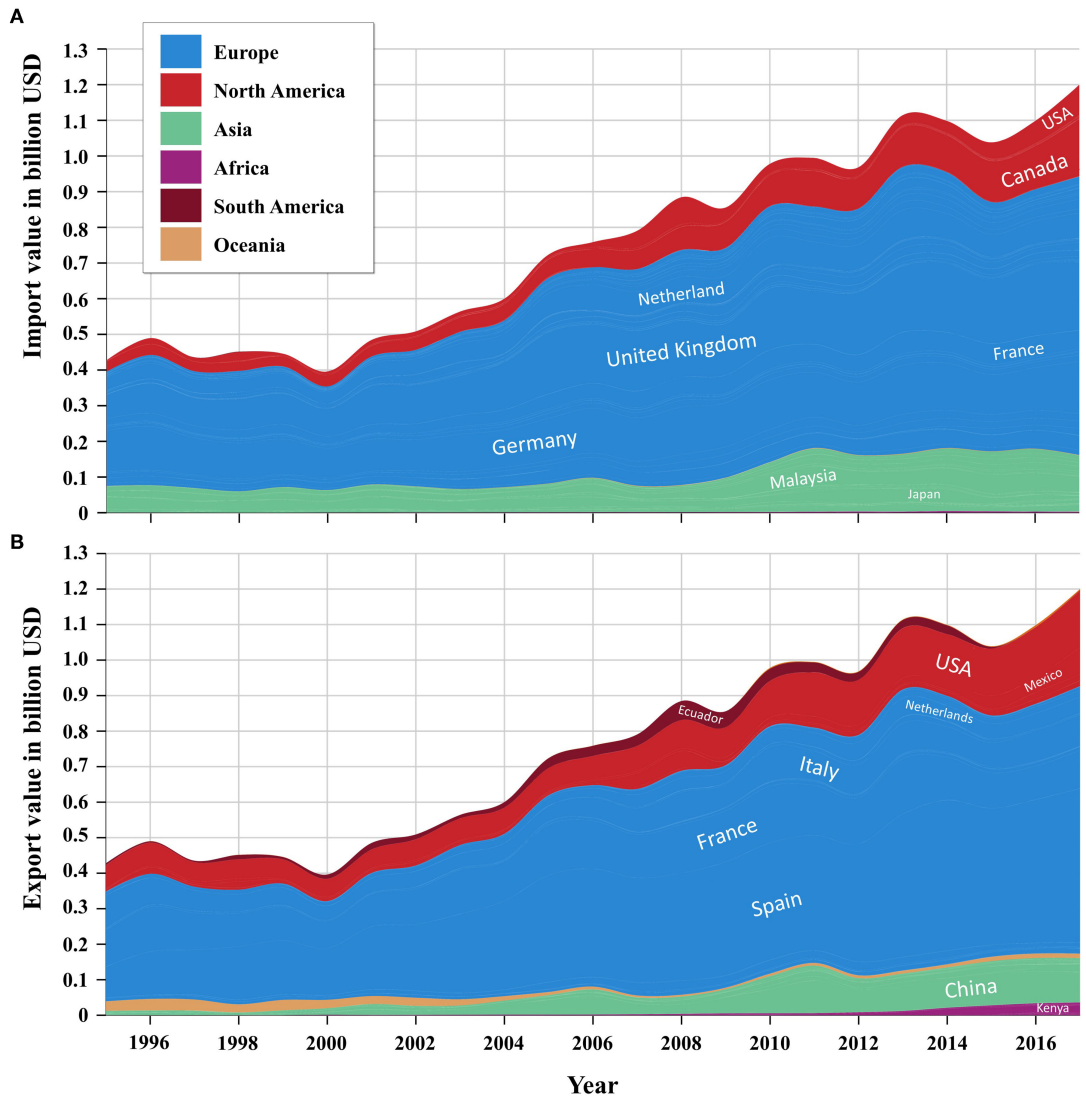
Global trend of broccoli and cauliflower import **(A)** and export **(B)** by countries from 1999 to 2017.

Since then, and particularly in the last 20 years, the import and export of broccoli and cauliflower greatly rose to a value exceeding $1.2 billion USD (Figure 2), alongside a 940% increase in their consumption (The Atlas of Economic Complexity, https://atlas.cid.harvard.edu). Market expansion was primarily driven by a growing global awareness over health and the ecological concept of “green living”; fresh and processed broccoli are, indeed, regularly identified among the vegetables habitually eaten to improve health and well-being (7).

Currently, the land cultivated with broccoli and cauliflower worldwide exceeds 1.4 million hectares (ha), with a yield of ~26 million metric tons that has been rising steadily over the years (+14.7% between 2012 and 2017). The top supplying countries are China and India, contributing about 40 and 33%, respectively, to the global production (8). The UK ranks first among importing countries, with a value of ~$210 million USD, followed by Canada, the USA, and Germany (UN Comtrade Database, https://comtrade.un.org/data).

Broccoli is marketed as either a fresh or processed product; however, the fresh market is considerably greater since the quoted price of broccoli for processing is typically 30–60% lower. Fresh broccoli also includes value added through minimally processed products, such as fresh-cut broccoli packed in bunches, bagged florets, and broccoli coleslaw—goods widely appreciated by a growing segment of health-conscious international consumers. Processed broccoli is typically frozen for retail sale and sold as spears or chopped, while only a limited amount is dehydrated and/or canned for ready-to-prepare mills and soups. In addition, the rising adoption of seed and sprout broccoli extracts in the food, cosmeceutical, and nutraceutical industries is estimated to account for significant revenue share in the global market, especially in the emerging economies of the Asia Pacific region. The global broccoli extract market was valued at over 1.9 billion US dollars in 2017 and is expected to reach a valuation in excess of $2.8 billion USD by the end of 2027, reflecting a compound annual growth rate (CAGR) of 3.7% during the forecast period (9).

## Broccoli as a Dietary Source of Bioactive Compounds

Broccoli heads have long been identified as a primary part of a well-balanced healthy eating plan. They are a low-calorie vegetable [34 kcal (~142 kJ)/100 g fresh weight (fw)] and a rich dietary source of minerals (calcium, phosphorus, potassium, and sodium), vitamins (B, C, E, K), fibers, and many other health-promoting molecules, including carotenoids (β-carotene and lutein), flavonoids (kaempferol), hydroxycinnamic acids (sinapic and caffeoyl-quinic acid derivatives), and distinctively, glucosinolates (GLs) (10, 11).

Compared with other organs (main stalk, leaves, and roots), edible broccoli heads are particularly rich in vitamin C (~188 mg/100 g fw) and are associated with a high content of polyphenols (64.9 mg/100 g fw) (12). Nevertheless, strong epidemiological evidence associates most of the positive health effects of broccoli consumption to the presence of GLs and their cognate breakdown products, isothiocyanates (13).

GLs represent 0.2–2% of broccoli heads' dry weight (dw) and comprise predominantly 4-methylsulphinylbutyl-GL (23–64%), followed by 3-indolylmethyl-, 4-hydroxy-3-indolylmethyl-, 3-methylsulphinylpropyl-, 1-methoxy-3-indolylmethyl-, and 4-methoxy-3-indolylmethyl-GL. Traces (<0.5 mg/g dw) of 4-methylthiobutyl-GL, 2(S)-2-hydroxy-3-butenyl-GL, 5-methylsulphinylpentenyl-GL, 2-hydroxy-4-pentenyl-GL, and 2-phenylethyl-GL have also been detected (14, 15). However, there is substantial variation regarding the total amount of GLs and the individual components among samples depending on several genetic, physiological, and environmental determinants (16).

GLs and isothiocyanates are potent anti-carcinogenic agents and widely recognized as effective inductors of phase II antioxidant enzymes (17). Particularly interesting in this regard is sulforaphane (4-methylsulfinylbutyl-isothiocyanate), a 4-methylsulphinylbutyl-GL-derived product of which broccoli represents the richest source among *Brassica* vegetables (13, 18, 19). 4-methylsulfinylbutyl-isothiocyanate inhibits chronic inflammatory processes and plays a multifaceted role in the onset, progression, and pleiotropic invasion of lung, stomach, colon, prostate, and rectal cancers (20–22). It upregulates the expression of some cytoprotective genes encoding for NAD(P)H:quinone oxidoreductase 1 (NQO1), heme oxygenase-1 (HO-1), thioredoxin, and superoxide dismutase (SOD), equilibrating ROS imbalance and inhibiting the expression of several pro-inflammatory mediators (13, 23, 24). Preclinical and clinical studies on animals (rats) and humans (healthy women undergoing breast reduction) provided evidence of a pronounced pharmacodynamic action of oral 4-methylsulfinylbutyl-isothiocyanate administration. A single dose of broccoli sprout preparation (150–200 μmol 4-methylsulfinylbutyl-isothiocyanate) prompted a significant upregulation of the expression of NQO1 and OH-1 transcripts, as well as an increase in NQO1 enzymatic activity. An improved bronchoconstriction with increased NQO1 expression was also observed in 60% of asthmatic patients following the daily oral intake (2 weeks) of 440 mg myrosinase-treated powdered broccoli sprouts extract (equivalent to 100 μmol 4-methylsulfinylbutyl-isothiocyanate) (13, 25). 4-methylsulfinylbutyl-isothiocyanate intake (~420 μmol daily dose in the form of 70 g broccoli sprouts) appears to significantly lessen or completely eradicate *Helicobacter pylori* infection with a concomitant decrease in gastric lumen inflammation of treated patients during the 8-weeks intervention period (26). Furthermore, a strong correlation between high serum 4-methylsulfinylbutyl-isothiocyanate concentration and (1) reduced fasting glucose levels, (2) stabilization of insulin, and (3) insulin resistance indices was reported in type 2 diabetes patients (27, 28). In addition, the intake of hot aqueous broccoli sprouts extracts (125 mL dose−5.2 μmol/mL of isothiocyanates after myrosinase hydrolysis) prompted the excretion of environmental carcinogens (dithiocarbamates) with a rate of 49 mmol/12 h (29). *In vitro* and *in vivo* trials are currently ongoing to assess the efficacy of 4-methylsulfinylbutyl-isothiocyanate intake on several other chronic and degenerative disorders including aging, allergic diseases, adverse drug reactions, cystic fibrosis, and gastrointestinal and smoke-related diseases (13).

Other GLs and isothiocyanates (e.g., 2-phenethyl isothiocyanate) demonstrated preventive properties against cancers and the ability to inhibit the metabolic activation of various carcinogens (30). Following BroccoMax® [a commercial broccoli supplement in pills (Jarrow Formulas, Los Angeles, CA, USA) containing a combination of 121 and 40 μmol 4-methylsulphinylbutyl-GL and 4-methylthiobutyl-GL, respectively, and active myrosinase] consumption, a decrease in ductal carcinoma cell proliferation, with an inverse association of the tumor marker Ki-67, was observed in patients with abnormal breast mammograms (13, 31). Megna et al. (32) reported a novel molecular activity of indole-3-carbinol in combatting various colorectal cells through cytotoxic and pro-apoptotic effects and activation of the aryl hydrocarbon receptor pathway. Quirit et al. (33) demonstrated that indole-3-carbinol prevents human melanoma cell growth by inhibiting NEDD4-1. Dekić et al. (34) found that phenylpentyl isothiocyanates exhibited cytotoxic effect on Caco-2 and HeLa cancer cell lines with 100-fold higher potency than papaverine. Núñez-Iglesias et al. (35) reported a dose-dependent cytotoxic effect of 3-butenyl isothiocyanates on prostate cancer.

However, not all GLs are equal in their biological effects, which rather depend on the specific amount and profile within a vegetable. Thus, a high *Brassica* consumption cannot be generally regarded as cancer-preventive (36). Recent *in vitro* and *in vivo* studies on mammalian cell cultures, bacteria, and animals indicated that broccoli and its extracts might also have adverse consequences for health, including mutagenic and genotoxic activities through the formation of characteristic DNA adducts. Adduct formation appears primarily prompted by the indole GLs [1-methoxy-3-indolylmethyl-GL, 4-methoxy-3-indolylmethyl-GL, and sinalbin (not detected in broccoli)] and requires the presence of myrosinase or gut microbiota thioglucosidase activity, suggesting a causal involvement of their breakdown products (37, 38). However, the relevance of the genotoxic activities to human health is not known yet (16).

Provided the importance of GLs in broccoli quality and human health, several reviews have dealt with various aspects of their chemistry and biosynthesis (20, 39–42), physiologic function in plant (43), and biological activity (13). The effect of post-harvest procedures and processing on GLs levels, as well as the influence of whole supply chain on intake, bioavailability, and human health associated with major *Brassica* vegetables consumption, were also appropriately reviewed (19, 44–46). However, a systematic summary on the effect of pre- and post-harvest factors/treatments on the levels and profiles of GLs in a large number of broccoli genotypes has never been carried out. Here, a broad overview of the published reports on this subject trying to fill the gap is provided. Despite the awareness given by the amount of recent research on GLs in broccoli, it is far from being comprehensive.

## GLs Chemical Structure, Metabolism, and Regulation in *Brassica*

**Figure 3:**
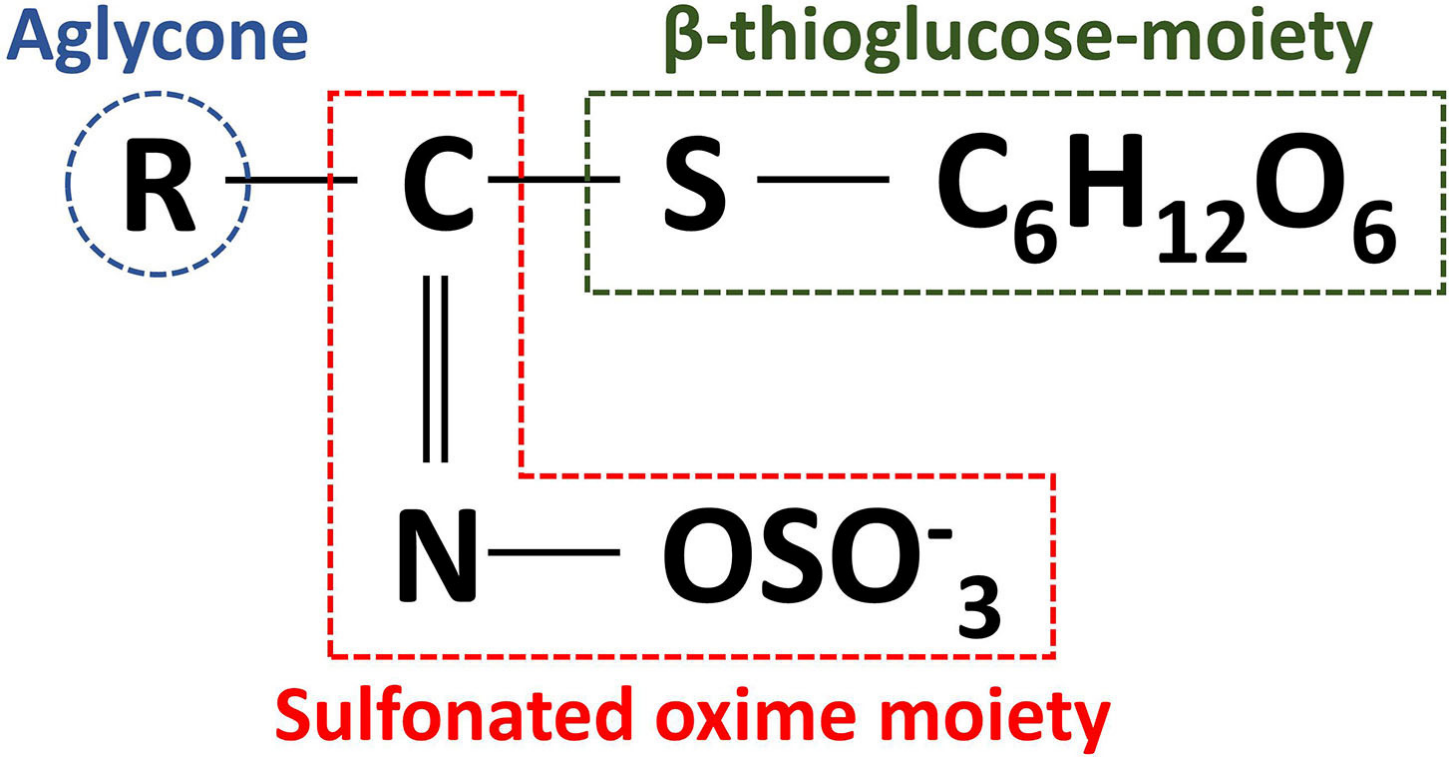
General structure of glucosinolates.

GLs are cis-N-hydroximinosulfate esters (S-glucopyranosyl thiohydroximates) naturally occurring as S-bound glucosides and consist of a β-thioglucose-moiety, a sulfonated oxime moiety, and a flexible aglycone α-amino acid-deriving side chain (Figure 3) (19, 20, 47). The level of GLs and their composition vary considerably not only between different *Brassica* crops, but also between different tissues within the same crop (48). More than 17 different GL have been identified in broccoli, classified as aliphatic (derived from the amino acids methionine, alanine, valine, leucine, or isoleucine), indole (derived from tryptophan), and aromatic GLs (derived from phenylalanine or tyrosine) (49).

**Figure 4:**
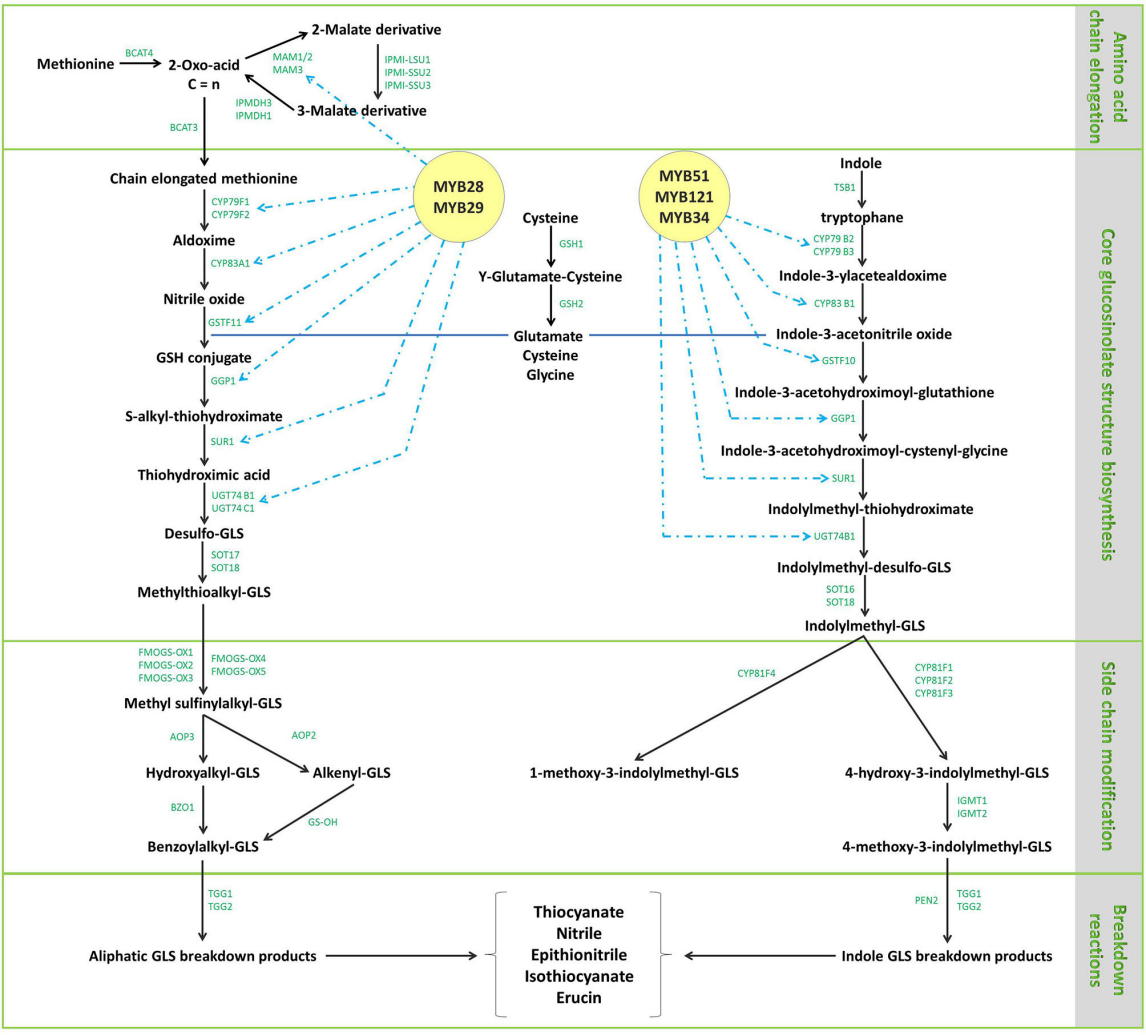
The aliphatic and indole glucosinolate metabolic pathways and related genes in Brassica oleracea L. Amino acid chain elongation is the first step, followed by the biosynthesis of the core glucosinolate structure and finally, side chain elongation of the synthesized structure in parallel with secondary modifications. The enzymes implicated in the different steps are reported in green. *MAM1-3*, methylthioalkyl malate synthases; *BCAT3/4*, branched-chain aminotransferases; *IPMDH1/3*, isopropylmalate dehydrogenases; *IPMI-SSU1/2/3*, isopropylmalate isomerases; *CYP79B2/3, CYP79F1/2, CYP81F1-4*, CYP83B1 cytochrome P450 monooxygenases; *GSTF10/11*, glutathione S-transferases; *CYP83A1*, non-redundant cytochrome P450. Enzymes metabolizing oximes in the biosynthesis of glucosinolates; *GGP1*, γ-glutamyl peptidase, *SUR1*, tyrosine transaminase family protein; *UGT74B1/C1*, thiohydroximate S-glucosyltransferases; *SOT16-18*, sulfotransferases; *FMOGS-OX1-5*, flavin-containing monooxygenase; *AOP2/3*, 2-oxoglutarate-dependent dioxygenases; *BZO1*, benzoyl-CoA ligase; *GS-OH*, 2-oxoglutarate (2OG) and Fe(II)-dependent oxygenase superfamily protein; *TGG1/2*, myrosinases; *TSB1*, tryptophan synthesis gene; *SUR1*, C-S lyase SUPERROOT; *UGT74B1*, UDP-dependent glycosyl transferases; *IGMT1/2*, indole glucosinolate methyl tansferases; *EN2*, atypical myrosinase; *GSH*, γ-glutamylcysteine synthetase; *MYB28/29/34/51/121* and *R2R3*, transcription factors.

**Figure 5:**
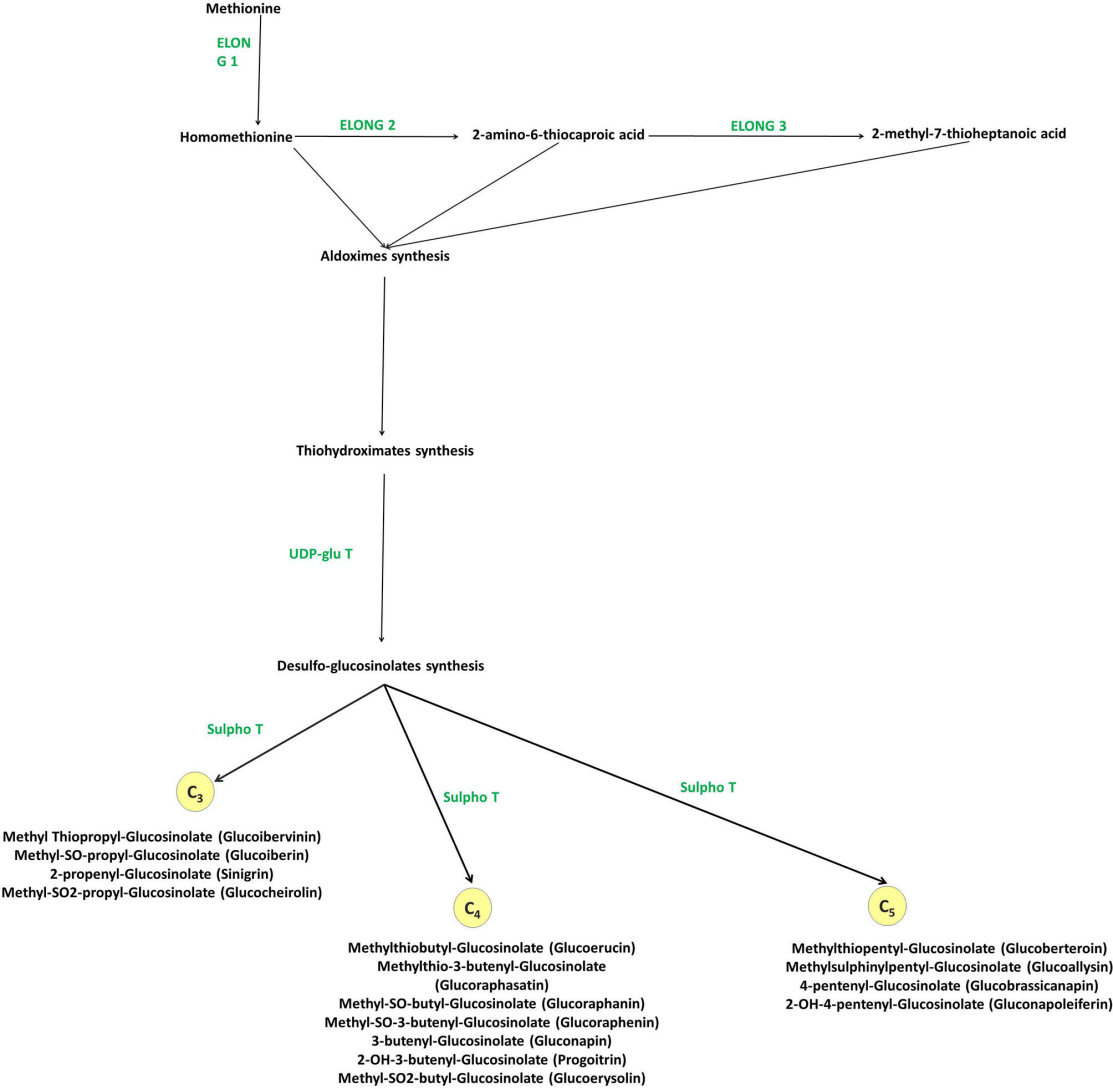
Methionine derived aliphatic GLs biosynthesis in *Brassica* vegetables. *Elong*, methionine elongation enzyme; *UDP-glu T*, UDP-glucose thiohydroximate glucotansferase; *Sulfo T*, 3′PAPS-5′-phosphosulphate desulphoglucosinolate transferase.

**Figure 6:**
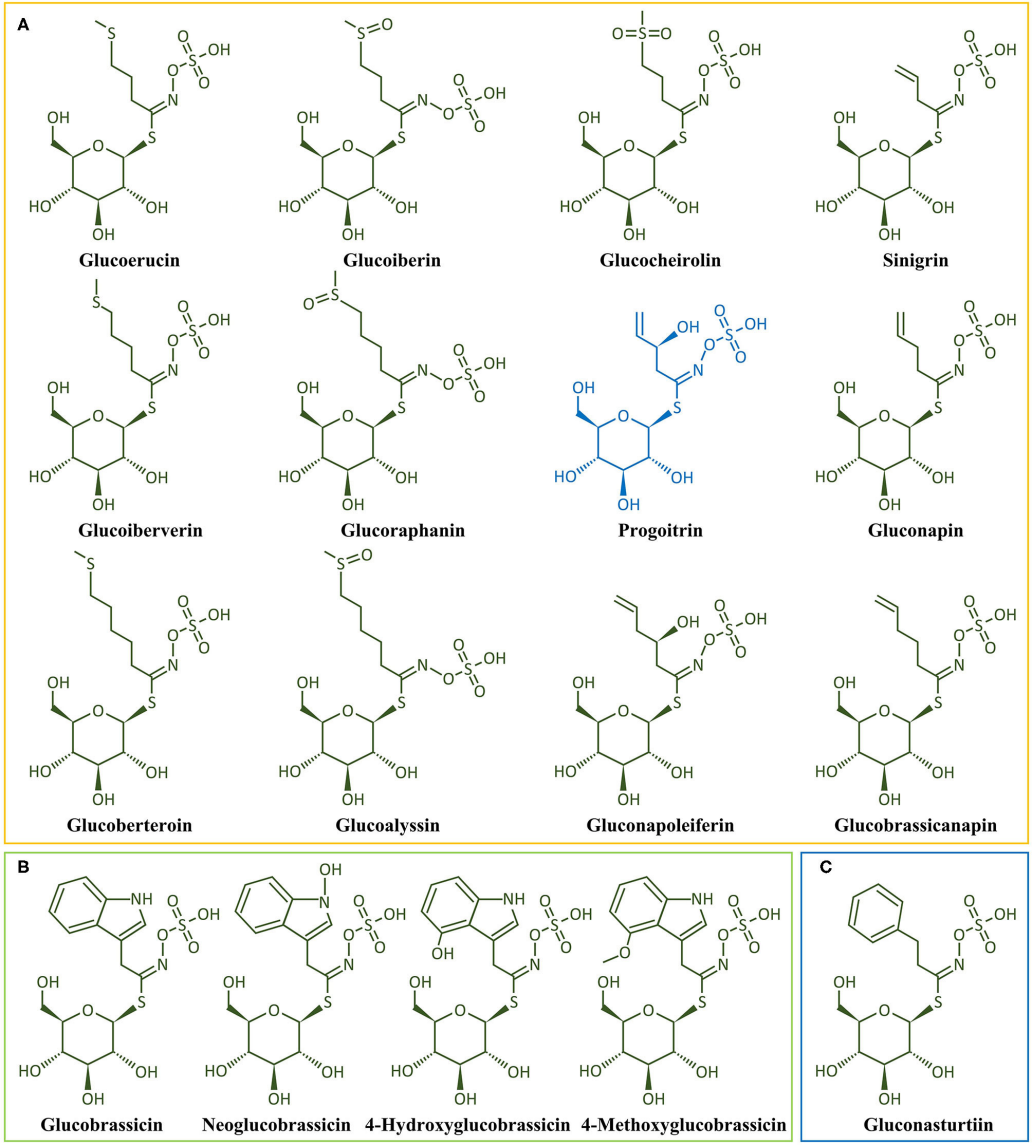
Chemical structure of the main glucosinolates found in broccoli. **(A)** Aliphatic Gls, **(B)** Indole GLs, and **(C)** Aromatic GLs. *Glucoerucin*, 4-Methylthiobutyl-GL; *Glucoiberin*, 3-Methylsulphinylpropyl-GL; *Sinigrin*, 2-Propenyl-GL; *Glucoibervirin*, 3-Methylthiopropyl-GL; *Glucoraphanin*, 4-Methylsulphinylbutyl-GL; *Progoitrin*, 2(R)-2-Hydroxy-3-butenyl-GL; *Gluconapin*, 3-Butenyl-GL; *Glucocheirolin*, 3-Methylsufonylpropyl-GL; *Glucoberteroin*, 5-Methylthiopentyl-GL; *Glucoalyssin*, 5-Methylsulphinylpentenyl-GL; *Gluconapoleiferin*, 2-Hydroxy-4-pentenyl-GL; *Glucobrassicanapin*, 4-Pentenyl-GL; *Glucobrassicin*, 3-Indolylmethyl-GL; *Neoglucobrassicin*, 1-Methoxy-3-indolylmethyl-GL; *4-Hydroxyglucobrassicin*, 4-Hydroxy-3-indolylmethyl-GL; *4-Methoxyglucobrassicin*, 4-Methoxy-3-indolylmethyl-GL; *Gluconasturtiin*, 2-Phenylethyl-GL.

The biosynthetic pathway of GLs has been thoroughly described in previous reviews (20, 39, 49). Aliphatic GLs are biosynthesized following three key independent steps: (1) the side chain elongation, (2) the GLs core structure building, and (3) the amino acid side chain modification (**>Figure 4**). Aliphatic GLs are characterized by the presence of a side chain of variable length shaped during the first step. Here, a non-polar amino acid, typically methionine, is deaminated to the corresponding 2-oxo-acid by a branched-chain amino acid aminotransferase (BCAT). Then, a sequential addition of single methylene groups (–CH2–) to the side chain of the 2-oxo-acid proceeds through a cycle of three consecutive enzymatic reactions. The reactions involve methylthioalkylmalate synthase (MAM), an isopropylmalate isomerase (IPMI), and isopropylmalate dehydrogenase (IPM-DH), yielding an elongated 2-oxo-acid. The elongated 2-oxo-acid can either proceed through another elongation round or be transaminated by a BCAT to the corresponding chain-elongated amino acid. Unlike indole GLs, a similar step of chain elongation is required for aromatic GL biosynthesis. Subsequently, cytochromes P450 (CYP79s) catalyzes the conversion of the amino acid derivatives to aldoximes, oxidized later by CYP83s into nitrile oxides, transformed to thiohydroximates via glutathione (GSH) S-transferases (GSTFs) and the C-S lyase (SUR1) reaction, and finally converted to the GL core structure by S-glucosyltransferases (UGT74s) and sulfotransferases (SOTs) (Figures 3–5). Simultaneously, GSH is produced by the action of specific ligases (GSH1 and GSH2) from cysteine, glutamate, and glycine (Figure 4, central part) and ensures sulfur supply for GL biosynthesis, yielding GSH conjugates (50, 51). Alkenylation, benzoylation, glycosylation, hydroxylation, methoxylation, oxygenation, and sulfonation may affect the elongated amino acid, increasing the chemical structure diversity of GLs (20, 39, 49). The methylthioalkyl GLs are S-oxygenated in a reaction catalyzed by flavin monooxygenases (FMOGS-OXs) to methylsulfinylalkyl GLs, such as 4-methylsulfinylbutyl-GL (Figure 6). GLs are usually stored in the vacuoles of specific laticifer-like sulfur-rich cells (S-cells) located between the endodermis and phloem along the vasculature and the leaf margins, ensuring the spatial separation of GLs from the enzymes involved in their hydrolysis (42).

**Figure 7:**
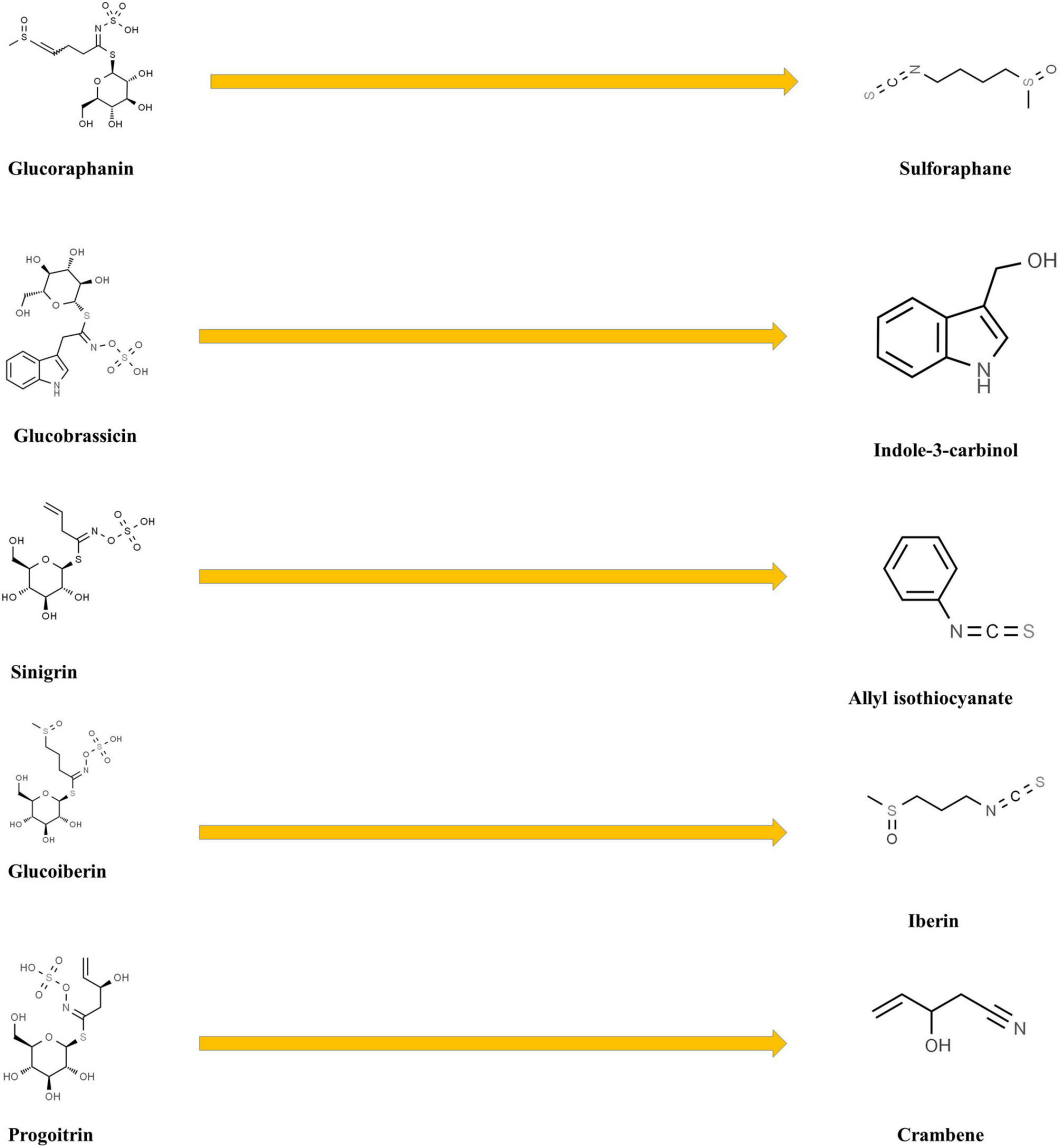
Breakdown products of some broccoli glucosinolates.

GL hydrolytic catabolism occurs through the action of myrosinases, specific β-thioglucoside glucohydrolases generating various bioactive products (isothyocyanates, mainly 4-methylsulfinylbutyl isothiocyanate, indole-3-carbinol, allyl isothiocyanate, crambene, iberin, and phenyl isothiocyanate, Figure 7) with recognized nematocidal, fungicidal, and insecticidal activities (52, 53) and controlling key biological process, such as abscisic acid (ABA)-mediated stomatal aperture (54), water regulation under sulfur deficiency (43), jasmonic acid (JA) signaling feedback regulation (55), growth responses (56, 57), flowering (58, 59), and defense reactions against pathogens and herbivores, suggesting a crosstalk with the regulatory networks controlling the developmental and physiological processes (60).

In *B. oleracea*, 105 genes distributed across the nine chromosomes are putatively related to GL biosynthesis, catabolism, and regulation, with the highest number of loci implicated in aliphatic and indole GL biosynthesis mapping on chromosomes 3 and 4, respectively (61, 62). The genes related to GL enzymatic breakdown, such as *PEN* and *TGG1*, and two encodes for β-thioglucoside glucohydrolases are able to cleave the indole (63) and aliphatic GLs, respectively (64). Specifier proteins, such as nitriles specifier proteins (NSPs), epithiospecifier proteins (ESPs), and thiocyanates specifier proteins (TSPs) affect the final breakdown products by inhibiting the synthesis of isothiocyanates in favor of simple nitriles, epithionitriles, and organic isothiocyanates, respectively (65–67).

The biosynthesis of GLs is finely tuned at transcriptional and hormonal levels. Several transcription factors have been identified: IQD1, a gene encoding a calmodulin-binding nuclear protein, and AtDoF1.1, a zinc finger protein, upregulate the biosynthesis of both aliphatic and indole GLs (42, 68, 69); SLIM1, an EIN3-like transcription factor, downregulates the expression of GLs biosynthetic genes (42, 70). Transcription factors, such as MYB28, MYB29, and MYB76 affect specifically the biosynthesis of aliphatic GLs, whereas MYB34, MYB51, and MYB122 are specific for indole GLs (Figure 4). R2R3-MYBs synergize with bHLH05 and bHLH06 to regulate the synthesis of GLs. However, the complex interaction bHLH06/MYC2 was found to negatively regulate the indole GLs biosynthetic pathway. Brassinosteroids (BRs), JA, salicylic acid (SA), and ethylene are known regulators of GL biosynthesis. Ethylene and SA positively affects the synthesis of all GL classes, while ABA is specific for the indole GLs (70). BRs were reported to downregulate CYP79B2 and the biosynthesis of aliphatic- and indole-GL. JA favors the degradation of the transcriptional repressor Jasmonate ZIM domain, which enables the transcription machinery access (42, 71).

## Factors Affecting GLs Synthesis and Accumulation

The content and profiles of GLs in broccoli are highly variable and affected by several factors. Genotype is certainly one of the major sources of variability; however, many other pre- and post-harvest aspects often interact, producing substantial effects on the health-promoting properties of broccoli. Although this aspect is central to cultivar recommendation for cancer chemoprevention and raises concerns about the synthesis of compounds reducing broccoli palatability, it also confers prospects for enhancing GL accumulation with no need for metabolic engineering approaches. Thus, it is of great relevance not only for breeders focusing on the development of high-quality genotypes, but also for processors and consumers (72).

### Genotypic Variability

Genotypic differences are known to significantly affect the content of many secondary metabolites in broccoli, including GLs and their breakdown products (73). Studying the genetic and environmental effects on GL content in the heads of various commercial broccoli cultivars (Everest, Futura, High Sierra, Viking, Marathon), new experimental lines (USVL), and their crosses, Farnham et al. (74) reported a large variability (up to 7.7-fold) in the content of 4-methylsulfinylbutyl-GL, the main aliphatic GL in broccoli florets. This was primarily ascribed to genotypic differences rather than environmental factors (≈22% of the total variance). Instead, genotypic effects were not significant for the concentrations of 4-hydroxy-3-indolylmethyl-, 1-methoxy-3-indolylmethyl, and indole-3-yl-methyl-GL. Even greater variability in the concentration of total GLs (99-fold change) and 4-methylsulfinylbutyl-GL (95-fold change) was reported by Bhandari and Kwak (75), comparing twelve broccoli cultivars (05-C3, AMaGi, BaeRiDom, CheongJae, Diamond, Grace, Grandeur, JikNok No. 28, NokJae, NokYeom No. 1, TS-2319, and YuDoRI No. 1) and reviewed thoroughly in Verkerk et al. (44). Essentially, 60% of aliphatic GLs appear more influenced by genotypic differences than environmental factors, while indole GLs appear concurrently affected by genotype, environmental conditions, and their interaction (44, 74, 76). Consequently, important advances were achieved in breeding for high aliphatic GL content (methionine-derived GLs). However, programs targeting specific indole GLs (tryptophan-derived GLs) appears more problematic (44). Wang et al. (77) focused on the natural variation of GLs in different broccoli genotypes grown under greenhouse conditions. The authors reported variation of 3.8- to 402-fold in the content of 4-methylsulphinylbuty-GL, 26- to 48-fold in the content of 3-indolylmethyl-GL, and 17.7- to 254.3-fold in the content of 1-methoxy-3-indolylmethyl-GL among commercial cultivars and their parental pure lines, respectively. This suggests the possible use of selected lines characterized by higher levels of beneficial GLs in breeding programs. Concurrently, the development of high GLs broccoli cultivars has been successfully achieved via the introgression of genetic material from *B. villosa* into an ordinary cultivar, with the resulting hybrids accumulating at least 3-fold more 4-methylsulfinylbutyl isothiocyanate than the parental counterpart. In purple broccoli cultivars, the content of 4-methylsulphinylbutyl-GL and 3-methylsulphinylpropyl-GL attained 6.7 and 3.8 mg/100 g fw, respectively (78). Sahamishirazi et al. (79) detected six major GLs in broccoli florets (3-methylsulfinylpropyl-GL, 2-propenyl-GL, 4-methylsulfinylbutyl-GL, indole-3-yl-methyl-GL, 4-methoxy-3-indolylmethyl-GL, and 1-methoxy-3-indolylmethyl-GL). 4-methylsulfinylbutyl-GL (0.03–2.87 μmol/g dw) and indole-3-yl-methyl-GL (0.21–0.73 μmol/g dw) contributed up to 34 and 17% to the total GLs amount, respectively, followed by 4-methoxy-3-indolylmethyl-GL, 1-methoxy-3-indolylmeth-GL, 2-propenyl-GL, and 3-methylsulfinylpropyl-GL, whose concentrations ranged between 0.43 and 0.12 μmol/g dw. The average of total, indole, and aliphatic GLs was 3.27, 1.59, and 1.56 μmol/g dw, respectively. Nicoletto et al. (80) performed a nutritional characterization of five Italian underutilized broccoli landraces (BF, BF4, BFT, BB, and BS) and revealed that BF4 accumulated 95% more 4-methylsulfinylbutyl isothiocyanate than the other lines. Genotypic differences were also evidenced in seeds and sprouts of three commercial broccoli cultivars (Marathon, Nubia, and Viola). 4-methylsulfinylbutyl-GL was the main aliphatic GL in sprouts of Nubia and Marathon but not in Viola, in which 3-methylsulfinylpropyl-GL prevailed. 4-methylsulfinylbutyl-GL ranged from 118.7 to 327.3 mg/100 g fw and from 26.3 to 204.1 mg/100 g fw in seeds and sprouts, respectively, and was more affected by genotype than 4-hydroxy-3-indolylmethyl-GL (142.2–167.9 and 2.3–13.5 mg/100 g fw in seeds and sprouts, respectively) (72, 73). In a fractionate analysis of GL profiles of several commercial and novel broccoli cultivars, 4-methylsulfinylbutyl-GL, 2(R)-2-Hydroxy-3-butenyl-GL, 4-methylthiobutyl-GL and indole-3-yl-methyl-GL were found to prevail in both flowers stalks and florets (81). Some hybrid lines (5,075, 5,078, and 5,079) and the inbred line 5,308 accumulated considerable levels of 4-methylsulfinylbutyl-GL, while other inbred lines (5,307, 5,311, and 5,409) accumulated higher levels of indole-3-yl-methyl-GL, underlying their promising use in further breeding programs to enhance broccoli nutritional quality. Bhandari and Kwak (75) compared the GL profiles and concentrations in the seeds, sprouts, mature roots, and shoots of broccoli. The total GL content was considerably higher in seeds and sprouts (110.8 and 162.2 μmol/g dw) compared to shoots and roots fractions (4.2 and 48.5 μmol/g dw). (2R)-2-Hydroxy-3-butenyl-GL and 4-methylthiobutyl-GL were mostly abundant GLs in seeds and sprouts accounting for 17–27 and 67–76% of their total content, respectively. Rybarczyk-Plonska et al. (82) reported that broccoli buds had 2.61-, 2-, and 12-fold higher concentrations of indole-3-yl-methyl-GL and 1-methoxy-indole-3-yl-methyl-GL contents than stalks with similar concentrations of total aliphatic GLs in all organs. Formica-Oliveira et al. (83) noticed that broccoli leaves presented 2.5/14.5 higher 4-methylsulfinylbutyl-GL/indole-3-yl-methyl-GL ratio compared to florets.

### Pre-harvest Factors

Several pre-harvest factors have demonstrated to be effective in altering the synthesis, accumulation, and profile of GLs in broccoli. These include the developmental stage at harvest, environmental, and seasonal variations, and most agricultural practices (irrigation, fertilization, salinity, priming, and elicitation). An overview of the current state of research and key results is given in Table 1.

**Table 1:** The effect of pre-harvest manipulations on the level of broccoli glucosinolates (GL class, individual GLs, and/or GL biosynthetic enzymes).

**Pre-harvest manipulations**	**Targeted GLS class/Individual GLS/GLS Biosynthetic enzymes**	**Cultivar name and/or particular specifications**	**Effect on GLS class/Individual GLS/GLS Biosynthetic enzymes**	**References**
**GENOTYPIC VARIABILITY**				
	Glucoraphanin content	Cvs Everest, Futura, High Sierra, Viking, Marathon, USVL049, USVL073, USVL046, USVL036, USVL048, USVL069, USVL066, USLVL032, USVL042, USVL045, USVL047, USVL022, USVL075, USVL039, USVL009, USVL018, USVL076, USVL013, USVL029, USVL044, USVL028, USVL070, USVL016, USVL013 X USVL073, USVL018 X USVL039, USVL047 X USVL036, and USVL013 X USVL075	4-methylsulfinylbutyl-GL content ranged from 24.7 to 265.6 μmol/head and significant correlation with QR activity	(74)
	Quinone reductase activity		Quinone reductase activity ranged from 1.32 to 12.43 unit/head 10^6^	
	Total GLs	NA	99-fold variation in total GL content	(44)
	4-Methylsulfinylbutyl-GL	Cvs 05-C3, AMaGi, BaeRiDom, CheongJae, Diamond, Grace, Grandeur, JikNok No. 28, NokJae, NokYeom No. 1, TS-2319, and YuDoRI No. 1	95-fold variation in 4-methylsulfinylbutyl-GL content	(75)
	Total aliphatic and indole GLs	2 broccoli F5 inbreds Ev6-1 (F6) and Eu8-1 (F6), 2 double haploids Su003 and VI-158, and 5 commercial hybrids cultivars, Baccus, Brigadier, High Sierra, Majestic, and Pirate	Total aliphatic GLs (60%) are more influenced by genotypic differences than environmental factors	(76)
		Cvs Everest, Futura, High Sierra, Viking, Marathon, USVL049, USVL073, USVL046, USVL036, USVL048, USVL069, USVL066, USLVL032, USVL042, USVL045, USVL047, USVL022, USVL075, USVL039, USVL009, USVL018, USVL076, USVL013, USVL029, USVL044, USVL028, USVL070, USVL016, USVL013 X USVL073, USVL018 X USVL039, USVL047 X USVL036, and USVL013 X USVL075	Indole GLs are affected by genotype, environmental conditions, and their interaction	(74)
		NA		(44)
	4-Methylsulphinylbuty-GL	Commercial cultivars and their parental pure lines	3.8- to 402-fold variation in the content of 4-methylsulphinylbuty-G	(77)
	4-Methylsulphinylbuty-GL		48- to 25.7-fold variation in the content of 3-indolylmethyl-GL	
	1-Methoxy-3-indolylmethyl-GL		17.7- to 254.3-fold variation in the content of 1-methoxy-3-indolylmethyl-GL	
	4-Methylsulfinylbutyl isothiocyanate	Wild ancestor *Brassica* villosa based hybrids	New hybrid accumulating at least 3-fold higher 4-methylsulfinylbutyl isothiocyanate content with respect to their standard counterparts	(78)
	4-Methylsulphinylbutyl-GL	Purple broccoli cultivars	4-methylsulphinylbutyl-GL and 3-methylsulphinylpropyl-GL content attained 6.7 and 3.8 mg/100 g fw, respectively	(44)
	3-Methylsulphinylpropyl-GL			
	4-Methylsulphinylbutyl-G	New bred open pollinating genotypes of broccoli	4-methylsulfinylbutyl-GL (0.03–2.87 μmol/g dw) and indole-3-yl-methyl-GL (0.21–0.73 μmol/g dw) contributed up to 34 and 17% to the total GLs amount, respectively, followed by 4-methoxy-3-indolylmethyl-GL, 1-methoxy-3-indolylmeth-GL, 2-propenyl-GL and 3-methylsulfinylpropyl-GL whose concentrations ranged between 0.43 and 0.12 μmol/g dw. The average of total, indole, and aliphatic GLs was 3.27, 1.59, and 1.56 μmol/g dw, respectively	(79)
	Indole-3-yl-methyl-GL			
	4-Methoxy-3-indolylmethyl-GL			
	1-Methoxy-3-indolylmeth-GL			
	2-Propenyl-GL			
	3-Methylsulfinylpropyl-GL			
	4-Methylsulfinylbutyl isothiocyanate	Five Italian underutilized broccoli landraces (Cvs BF, BF4, BFT, BB, and BS)	BF4 accumulated 95% higher 4-methylsulfinylbutyl isothiocyanate than the other lines	(80)
	4-Methylsulfinylbutyl-GL	Cvs Marathon, Viola, and Nubia	4-methylsulfinylbutyl-GL was the main aliphatic GL in sprouts of Nubia and Marathon 3-methylsulfinylpropyl-GL prevailed in Viola sprouts 4-methylsulfinylbutyl-GL ranged from 118.7 to 327.3 mg/100 g fw and from 26.3 to 204.1 mg/100 g fw in seeds and sprouts, respectively 4-hydroxy-3-indolylmethyl-GL ranged from 142.2 to 167.9 and from 2.3 to 13.5 mg/100 g fw in seeds and sprouts, respectively	(72)
	3-Methylsulfinylpropyl-GL	Cvs Iron Man, Marathon, and Sirtaki		(73)
	4-Hydroxy-3-indolylmethyl-GL			
	4-Methylsulfinylbutyl-GL, 2(R)-2-Hydroxy-3-butenyl-GL	Cvs SK3-085, Woosu, Neahanwoosu, Yeonmu, Very Dome, Ace Dome, Engmu, Kanghan, Koyosi, Supergrace, 19 F1 hybrids, and 20 inbred lines	4-methylsulfinylbutyl-GL, 2(R)-2-Hydroxy-3-butenyl-GL, 4-methylthiobutyl-GL and indole-3-yl-methyl-GL prevail in stalks and florets 5,075, 5,078, and 5,079 hybrids and the inbred line 5,308 accumulated considerable levels of 4-methylsulfinylbutyl-GL 5,307, 5,311, and 5,409 inbred lines accumulated higher levels of indole-3-yl-methyl-GL	(81)
	4-Methylthiobutyl-GL			
	Indole-3-yl-methyl-GL			
	Total GLs	Cv. Greendom	Total GL content was considerably higher in broccoli seeds and sprouts (110.8 and 162.2 μmol/g dw) compared to shoots and roots fractions (4.2 and 48.5 μmol/g dw)	(84)
	(2R)-2-Hydroxy-3-butenyl-GL		(2R)-2-hydroxy-3-butenyl-GL and 4-methylthiobutyl-GL were mostly abundant GLs in seeds and sprouts and accounted, respectively for 17–27 and 67–76% of their total content	
	4-Methylthiobutyl-GL			
	Indole-3-yl-methyl-GL	Cv. Marathon	Broccoli buds had 2.61-, 2- and 12-fold higher indole-3-yl-methyl-GL and 1-methoxy-indole-3-yl-methyl-GL contents than stalks	(82)
	1-Methoxy-indole-3-yl-methyl-GL			
	4-Methylsulfinylbutyl-GL	Bimi broccoli	Broccoli leaves presented 2.5/14.5 higher 4-methylsulfinylbutyl-GL/indole-3-yl-methyl-GL ratio compared to florets	(83)
	Indole-3-yl-methyl-GL			
**DEVELOPMENT STAGE AT HARVEST**				
	Total GLs	Cv. Saga	Young broccoli sprouts accumulate 20-fold higher GLs than late vegetative stages GLs levels progressively decrease with increasing sprouts maturity	(85)
		Cvs Marathon, Viola and Nubia		(72)
		Cvs Iron Man, Marathon and Sirtaki		(73)
	Total GLs	Cvs Marathon, Viola, and Nubia	Broccoli seeds show significantly higher content of total and aliphatic GLs (3-methylsulfinylpropyl-GL, 4-methylsulfinylbutyl- GL and 4-Hydroxy-3-indolylmethyl-GL) than 2-weeks old sprouts	(72)
	Aliphatic GLs			
	3-Methylsulfinylpropyl-GL			
	4-methylsulfinylbutyl- GL			
	4-Hydroxy-3-indolylmethyl-GL			
	Indole GLs		Broccoli seeds show lower levels of indole GLs (indole-3-yl-methyl-GL and 1-methoxy-3-indolylmethyl), suggesting that the two GL classes are differentially used as chemical defense system against aggressors during plant development	
	Indole-3-yl-methyl-GL			
	1-Methoxy-3-indolylmethyl			
	Aliphatic GLs	Cvs Iron Man, Marathon, and Sirtaki	Higher aliphatic GLs in sprouts being progressively replaced by indolic GLs in fully developed heads	(73)
	Total GLs		Young broccoli heads had the highest levels of total GLs and isothiocyanates rather than those at commercial stage	
	4-Methylsulfinylbutyl-GL	Cvs Claudia, Marathon, and TB-234	A linear increasing trend for 4-methylsulfinylbutyl-GL content throughout the development stages was instead in three broccoli	(86)
	4-Methylsulphinylbutyl-GL	The commercial cultivar “Koyoshi” and five new-variety candidates	Linear increase in 4-Methylsulphinylbutyl-GL	(87)
	3-Indolylmethyl-GL			
	2-Phenylethyl-GL		Decreasing trend for 3-Indolylmethyl-GL	
	3-Methylsulphinylpropyl-GL		Cultivar-dependent dynamic changes of 2-phenylethyl-GL	
	2(R)-2-Hydroxy-3-butenyl-GL		2-phenylethyl-GL peaked at the first stage of ripening in cultivar 12FA-M296 (0.55 μmol/g dw), at intermediate stage of ripening in cultivars 10FA-M806, 10FA-M853 12FA-M413 and Koyoshi with levels ranging (0.24–0.36 μmol/g dw) and at the commercial stage in cultivar 09FA-M295 (0.39 μmol/g dw)	
	3-Butenyl-GL		3-methylsulphinylpropyl-GL, 2(R)-2-hydroxy-3-butenyl-GL and 3-butenyl-GL were present throughout the ripening stages only in some genotypes	
	Total GL	Cultivars (Marathon, Monterrey, and Vencedor)	Under poor sulfur fertilization, total GLs peaked at the early stages of development 35 DAT in the cultivar Vencedor, and 42 DAT in Marathon and Monterrey	(88)
	4-Methylsulfinylbutyl-GL		4-methylsulfinylbutyl-GL peaked at 42 DAT in cultivar Vencendor, at 49 DAT in Marathon, and 56 DAT in Monterrey under different fertilization regimes	
	1-Methoxy-3-indolylmethyl-GL		1-methoxy-3-indolylmethyl-GL peaked at 35 DAT in Vencendor, 42 DAT in Marathon and 49 DAT in Monterrey	
	Indole-3-yl-methyl-GL		Indole-3-yl-methyl-GL peaked at 42 DAT in Monterry and Vencendor	
	4-Methoxy-3-indolylmethyl		4-methoxy-3-indolylmethyl peaked at 35 DAT in cultivars Marathon and Vencendor but at 49 DAT in Monterrey	
	4-Methylsulphinylbutyl isothiocyanate	NA	Linear increase in 4-methylsulphinylbutyl isothiocyanate content until full commercial maturity and declining after flower setting	(44)
**ENVIRONMENTAL AND SEASONAL VARIATION**				
	GL biosynthesis genes	NA	Light induces expression of GL biosynthesis genes Genotype-specific gene responses to temperature	(89)
	Total GLs	Cv.Marathon	Total GL content and their individual profile are modulated by the interaction temperature x photoperiod for field grown broccoli	(90)
		Cv. Lord		(14)
		Cv. Marathon		(91)
	4-Methylsulfinylbutyl-GL	Cv. Youxiu	The highest 4-methylsulfinylbutyl-GL and 4-methylsulfinylbutyl isothiocyanate were detected at 25°C	(92)
	4-Methylsulfinylbutyl isothiocyanate		At 20 or 30°C 4-methylsulfinylbutyl-GL and 4-methylsulfinylbutyl isothiocyanate content decreased during sprout growth	
	Aliphatic and indole GL biosynthetic enzymes	NA	Differential activation of aliphatic and indolic GL biosynthetic enzymes by light and temperature in *Brassica* leaves were higher at 32 and 12°C when compared to 22°C under constant lighting conditions Total and aliphatic GL content in broccoli sprouts were higher at 11 and 33°C than intermediate temperatures	(44)
	4-Methylsulfinylbutyl-G	Cv. Marathon	Under daily temperature range of 7–13°C and moderate daily mean radiation of 10–13 mol/m^2^/day, 4-methylsulfinylbutyl-GL and 3-methylsulphinylpropyl-GL increased 8-fold whereas 3-Indolylmethyl-GL was reduced	(91)
	3-Methylsulphinylpropyl-GL			
	3-Indolylmethyl-GL			
	4-Methylsulfinylbutyl-	Cv. Lord	Higher 4-methylsulfinylbutyl-GL levels at 12°C than at 18°C in the florets of broccoli grown under long photoperio	(14)
	3-Methylsulfinylpropyl-GL		Higher 3-methylsulfinylpropyl-GL levels at 18°C than at 12°C under short day	
	Total aliphatic GLs		33% higher total aliphatic GL content under long day at 12°C than 18°C	
	4-Hydroxy-indole-3-yl-methyl-GL		4-hydroxy-indole-3-yl-methyl-GL and 1-methoxy-indole-3-yl-methyl-GL were greater at 18°C, under short day than under long day photoperiod	
	1-Methoxy-indole-3-yl-methyl-GL			
	Total indole GLs		Total indole GL content increased by 24% at 18°C with respect to 12°C	
	4-Methylsulphinylbuty-GL	Cv. Lord	4-methylsulphinylbuty-GL was unaffected by variations in temperatures (15/9°C and 21/15°C)	(93)
	4-Methoxy-3-indolylmethyl-GL		4-methoxy-3-indolylmethyl-GL and indole-3-yl-methyl-GL were unaffected by day length (12 and 24 h)	
	Indole-3-yl-methyl-GL			
	Total GLs		High day/night temperatures were associated with peaks in total GLs (31.5–33.2 mg/g dw) regardless of the photoperiod	
	4-Methylsulfinylbutyl- isothiocyanate	Cvs Brigadier and Emperor	4-methylsulfinylbutyl isothiocyanate content was higher in fall harvests than in spring	(94)
	Total GLs	Cvs 05-C3, AMaGi, BaeRiDom, CheongJae, Diamond, Grace, Grandeur, JikNok No. 28, NokJae, NokYeom No. 1, TS-2319, and YuDoRI No. 1	The highest total GL content was measured in broccoli florets grown in spring (2.26–17.81 μmol/d dw) compared to fall season (3.17–13.8 μmol/d dw)	(75)
			In broccoli stems, higher total GL content was measured in fall (2.91–7.92 μmol/d dw) compared to spring season (1.04–5.74 μmol/d dw)	
	4-Methylsulfinylbutyl isothiocyanate	Cv. Parthenon	Greater 4-methylsulfinylbutyl isothiocyanate content in fall (32.2–99.9 mg/kg fw) than in spring (9.9–13.3 mg/kg fw) harvest	(95)
			Last-fall harvest showing 164–261% higher methylsulfinylbutyl- isothiocyanate compared to first-fall harvest depending on the applied treatments	
	Total and individual GLs	NA	Higher levels of total and individual GLs are measured in late (August–January) compared to early (April–July) cropping seasons	(44)
**AGRICULTURAL PRACTICES**				
	4-Methylsulphinylbuty-GL	NA	Low rainfall increased the levels of 4-methylsulphinylbuty-GL and methylsulphinylbuty-isothiocyanate in most of the *Brassica* vegetables	(44)
	Methylsulphinylbuty-isothiocyanate			
	Total GLs	Cvs Marathon, Monterrey, and Vencedor	Cultivar-dependent response on total GL content of marketable broccoli heads (56 DAT) under rich and poor sulfur fertilization	(88)
			Under rich sulfur supply, ~10% decrease and ~69% increase in total GL content, respectively in cultivars Monterrey and Vencendor, was associated with rich sulfur supply	
			Total GL content in cultivar Marathon remained statistically unchanged	
	4-Methylsulfinylbutyl-GL	Cvs Claudia, Marathon, and TB-234	50–70% increase in 4-methylsulfinylbutyl-GL content in florets and whole plant fractions (cultivar Marathon) under low (23 kg S/ha) and high (92 kg S/ha) sulfur supply regimes, respectively throughout maturity stages 4-methylsulfinylbutyl-GL content was more responsive to sulfur supply in cultivar Claudia than cultivar Marathon or TB-234.	(86)
	3-Butenyl-	Cv. Parthenon	60–80% decrease in 3-Butenyl-GL content with decreasing NO3^−^/NH4^+^	(96)
	Indole-3-yl-methyl-GL		Indole-3-yl-methyl-GL and 1-Methoxy-3-indolylmethyl-GL as well as total indole GLs significantly increased with decreasing NO3^−^/NH4^+^ ratio and elevated CO_2_ concentrations	
	1-Methoxy-3-indolylmethyl-GL			
	Total indole GLs			
	Total GLs	Cv. Belstar	The application of olive tree pruning based biochar increased GL content in broccoli florets with respect to conventionally fertilized treatments which registered the lowest concentration of 1-methoxy-3-indolylmethyl-GL and methylsulphinylbuty-GL	(97)
	1-Methoxy-3-indolylmethyl-GL			
	Methylsulphinylbuty-GL			
	3-Indolylmethyl-GL	NA	Organically grown broccoli exhibited significantly higher amounts of 3-Indolylmethyl-GL than their conventionally grown counterparts	(98)
	3-Indolylmethyl-GL	Cv. Groene Calabrese	240 and 6% higher 3-indolylmethyl-GL and 1-methoxy-3-indolylmethyl-GL content under conventional compared to organic farming	(99)
	1-Methoxy-3-indolylmethyl-GL			
	Total GLs	NA	An accumulation of GL content and overall improvement of the nutritional quality of broccoli florets under moderate salinity stress	(100)
	Total GLs	Cv. Marathon	Total GLs levels increased in broccoli florets disregarding the concentrations of NaCl applied (40 or 80 mM)	(101)
	Total GLs	Cvs Parthenon and Naxos	Opposite response among cultivars Parthenon and Naxos to salinity stress (0, 30, 60, and 90 mM NaCl)	(102)
			Total GLs significantly decreased in Naxos due to a sharp decline in the content of indolic GLs; it was practically unaffected	
	Indolic GLs		Total GLs remain unchanged in Parthenon	
	Methylsulphinylbuty-GL	Cv. Parthenon	Aliphatic GLs (methylsulphinylbuty-GL and 4-methylthiobutyl-GL) increased in plant grown under salinity stress	(43)
	4-Methylthiobutyl-GL			
	4-Hydroxy-3-indolylmethyl-GL 3-Indolylmethyl-Gl		Indolic GLs (4-hydroxy-3-indolylmethyl-GL, 3-indolylmethyl-Gl, 4-methoxy-3-indolylmethyl-GL and 1-methoxy-3-indolylmethyl-GL) decreased in plant grown under salinity stress	
	4-Methoxy-3-indolylmethyl-GL 1-Methoxy-3-indolylmethyl-GL			
	4-Methylsulfinylbutyl-GL	Cv. Marathon	4-methylsulfinylbutyl-GL and 4-methoxy-3-indolylmethyl-GL decreased in in cultivar Marathon by 61 and 74%, respectively 1 day following 80 mM saline treatment	(103)
	4-Methoxy-3-indolylmethyl-GL			
	Indole-3-yl-methyl-GL		Indole-3-yl-methyl-GL and 3-methylsulfinylpropyl-GL decreased by 65 and 70%, respectively, at day 6 compared to the control plants	
	3-Methylsulfinylpropyl-GL		Decreasing trend for all GLs between 40 and 80 mM followed by a slight increase of all individual GLs except for 4-methoxy-3-indolylmethyl-GL	
	Total GLs	Cv. Marathon	Combination of salinity and foliar sprayed elicitors (methionine, tryptophan and chitosan) may increase the content of various secondary metabolites in broccoli florets under hydroponic growing conditions	(104)
	3-Indolylmethyl-GL	Cv. Olimpia	Indolic GLs (3-indolylmethyl-GL and 1-methoxy-3-indolylmethyl-GL) increased in broccoli florets irrigated with saline water (4 dS/m) from transplanting to appearance of the inflorescence	(105)
	1-Methoxy-3-indolylmethyl-GL			
	Total GLs		Total GLs remained unchanged in broccoli heads exposed to the same salinity stress from transplanting to harvest	
	4-Methylsulfinylbutyl-GL	NA	4-methylsulfinylbutyl-GL, indole-3-yl-methyl-GL, 4-methoxy-3-indolylmethyl-GL, and 1-methoxy-3-indolylmethyl-GL levels were, interestingly, increased in broccoli sprouts by 48–247% following 25 μM MeJA treatments, with 4-methylsulfinylbutyl-GL rising from 183 to 294 mg/100 g fw	(106, 107)
	Indole-3-yl-methyl-GL			
	4-Methoxy-3-indolylmethyl-GL			
	1-Methoxy-3-indolylmethyl-GL			
	Indole-3-yl-methyl-GL	NA	Indole-3-yl-methyl-GL, 1-methoxy-3-indolylmethyl-GL, 4-hydroxy-3-indolylmethyl-GL and 4-methylthiobutyl-GL, 3-methylsulfinylpropyl-GL, 3-methylthiopropyl-GL, and 4-methylsulfinylbutyl-GL content increased in microgreens treated with 10 mM CaCl_2_ Low concentration of 1 mM CaCl_2_ was found ineffective	(108)
	1-methoxy-3-indolylmethyl-GL	Cv. De Cicco		(109)
	4-hydroxy-3-indolylmethyl-GL			
	4-methylthiobutyl-GL			
	3-methylsulfinylpropyl-GL			
	3-methylthiopropyl-GL			
	4-methylsulfinylbutyl-GL			
	4-Methylsulfinylbutyl	Cv. Green Magic	Increase in 4-methylsulfinylbutyl isothiocyanate, neoascorbigen, N-methoxyindole-3-carbinol and phenethyl isothiocyanate, in broccoli fresh and stored florets subjected to pre-harvest application of 500 ppb MeJA and post-harvest treatments with 1-methylcyclopropene	(110)
	Isothiocyanate			
	Neoascorbigen			
	N-methoxyindole-3-carbinol			
	Phenethyl isothiocyanate			
	4-Methylthiobutyl-GL	Cv. Youxiu	50% increases in 4-methylthiobutyl-GL in 10 mM CaCl_2_ treated broccoli sprouts as compared to the control	(111)
	Total aliphatic GL	Cv. Parthenon	19% increase in total aliphatic GL content after K_2_SO_4_ application mainly ascribed to a peak in methylsulphinylbuty-GL and 765% increase in total indole GL content following MeJA foliar spray application. 99% decrease in indole-3-yl-methyl-GL levels occurred following all the treatments with the greatest decrease (99%) following KCl priming	(112)
	Methylsulphinylbuty-GL			
	Total indole GLs			
	Indole-3-yl-methyl-GL			
	4-Methylsulfinylbutyl-GL	Cvs Diplomat and Gypsy	50% decrease in 4-methylsulfinylbutyl-GL and 1-methoxy-3-indolylmethyl-GL were observed in 25 μM sodium selenate (Na_2_SeO_4_) treated florets	(113)
	1-Methoxy-3-indolylmethyl-GL			
	Aliphatic indole GLs	NA	UV-B radiation at doses ranging from 0.3 to 1 kJ/m^2^/d prompted an increase in the levels of both aliphatic and indole GLs of broccoli sprouts	(114)
	Total GLs	Cv. Naxos	An increase in total GL content was in microgreens treated with 10 mM CaCl_2_ and 0.18 Wh/m^2^ UVB	(115)

#### Development Stage at Harvest

Broccoli head development is an important factor in defining their phytochemical composition, as substantial changes arise at in this stage (72, 87, 88). However, such data is currently rather limited. Even so, considerable cultivar-to-cultivar variation in GL metabolism has been reported during broccoli development (Table 1). Young broccoli sprouts accumulate up to 20-fold higher GLs (or breakdown products) than late vegetative stages, and their levels progressively decrease with increasing sprout maturity (72, 73, 85). Broccoli seeds show significantly higher content of total and aliphatic GLs (3-methylsulfinylpropyl-GL, 4-methylsulfinylbutyl-GL, and 4-hydroxy-3-indolylmethyl-GL) than 2-weeks-old sprouts but lower levels of indole GLs (indole-3-yl-methyl-GL and 1-methoxy-3-indolylmethyl), suggesting that the two GL classes are differentially used as chemical defense system against aggressors during plant development (72). However, Hanschen and Schreiner (73) reported higher aliphatic GLs in sprouts being progressively replaced by indole GLs in fully developed heads. Additionally, the highest levels of total GLs and isothiocyanates were detected in young heads rather than at the commercial stage. A linear increasing trend for 4-methylsulfinylbutyl-GL content throughout the development stages was instead reported by Rangkadilok et al. (86) in three broccoli cultivars (Claudia, Marathon, and TB-234). In agreement with Rangkadilok et al. (86), Bhandari et al. (87) reported also a linear increase in 4-methylsulphinylbutyl-GL and a decreasing trend for 3-indolylmethyl-GL. However, the dynamic changes affecting 2-Phenylethyl-GL was cultivar-dependent. 2-Phenylethyl-GL peaked at the first stage of ripening in cultivar 12FA-M296 (0.55 μmol/g dw), at the intermediate stage of ripening in cultivars 10FA-M806, 10FA-M853, 12FA-M413, and Koyoshi with levels ranging from 0.24 to 0.36 μmol/g dw, and at the commercial stage in cultivar 09FA-M295 (0.39 μmol/g dw). Some GLs, such as 3-methylsulphinylpropyl-GL, 2(R)-2-hydroxy-3-butenyl-GL, and 3-butenyl-GL were present throughout the ripening stages only in some genotypes. Instead, conflicting results emerged at later stages of development depending on genotype and plant mineral nutrition. Vallejo et al. (88) found that, under poor sulfur fertilization, total GLs peaked at the early stages of development [35 DAT in the cultivar Vencedor (60.3 μmol/g dw) and 42 DAT in Marathon (64.7 4 μmol/g dw) and Monterrey (65.7 μmol/g dw)], but the dynamic of change of the individual GLs was complex and dependent, besides genotype, upon their chemical class. 4-methylsulfinylbutyl-GL peaked at 42 DAT in cultivar Vencendor (2.3 μmol/g dw), at 49 DAT in Marathon (1.8 μmol/g dw), and 56 DAT in Monterrey (1.9 μmol/g dw) under different fertilization regimes. The behavior was even more complex for indole GLs: 1-Methoxy-3-indolylmethyl-GL peaked at 35 DAT in Vencendor (42.1 μmol/g dw), 42 DAT in Marathon (55.0 μmol/g dw), and 49 DAT in Monterrey (49.2 μmol/g dw). Indole-3-yl-methyl-GL peaked at 42 DAT in Monterry and Vencendor with levels of 13.4 and 14.4 μmol/g dw, respectively. However, two peaks were detected at 42 and 49 DAT (15.1 and 15.4 μmol/g dw, respectively) in Marathon. Similarly, 4-methoxy-3-indolylmethyl peaked at 35 DAT in the cultivars Marathon and Vencendor (1 and 1.6 μmol/g dw, respectively), but at 49 DAT in Monterrey (2.5 μmol/g dw). Verkerk et al. (44) reported that 4-methylsulphinylbutyl isothiocyanate content increased until full commercial maturity and declining after flower setting.

#### Environmental and Seasonal Variation

Broccoli can grow under various climatic conditions and geographical areas; this suggests significant effects, not only on crop production potential, but also on various quality attributes of broccoli (aroma, texture, taste, etc.) and on the levels of their health-promoting compounds (14, 116). It has been reported that the dynamic change in GLs is tightly correlated with temperature and day length and, as a consequence, by seasonal variation. Light induces expression of GL biosynthesis genes, resulting also in diurnal rhythms, while transcriptional profiling revealed genotype-specific gene responses to temperature but only a limited correlation between GL-related gene expression and GL levels (89). The data on field-grown broccoli pointed out that total GL content and individual profile are rather modulated by the interaction temperature × photoperiod (14, 90, 91, 117). During broccoli sprouts growth, Guo et al. (92) detected the highest 4-methylsulfinylbutyl-GL and 4-methylsulfinylbutyl isothiocyanate at 25°C, but lower (20°C) or higher (30°C) temperatures decreased their levels in cultivar Youxiu. This fact might be ascribed to differential activation of aliphatic and indole GL biosynthetic enzymes by light and temperature, respectively (44). Similarly, Verkerk et al. (44) reported that total and aliphatic GLs in *Brassica* leaves were higher at 32 and 12°C compared to 22°C under constant lighting conditions. However, in broccoli sprouts, their content was higher at 11 and 33°C than intermediate temperatures. Schonhof et al. (118), studying the effect of light radiation and temperature on greenhouse broccoli (Emperor, Shogun and Marathon cultivars) florets grown under high irradiation dose of 8–16 mol/m^2^/day, detected a peak of 4-methylsulfinylbutyl-GL at low temperatures (6–12°C) with respect to high temperature range (16–22°C). However, a peak of indole-3-yl-methyl-GL content was detected at higher temperature range (16–22°C) and low irradiation dose (2 mol/m^2^/day). The levels of 2(R)-2-hydroxy-3-butenyl-GL, 2-hydroxy-4-pentenyl-GL, 1-methoxy-3-indolylmethyl-GL, 4-methoxy-3-indolylmethyl-GL, and 4-hydroxy-3-indolylmethyl-GL were not affected by temperature or radiation. However, those of 3-methylsulfinylpropyl-GL and 4-methylsulfinylbutyl-GL were considerably increased under low temperature and high irradiation (91). Conversely, Mølmann et al. (14) detected significantly higher 4-methylsulfinylbutyl-GL levels at 12°C than at 18°C in the florets of broccoli grown under long-day photoperiod, while the differences were non-significant under short-day photoperiod. 3-methylsulfinylpropyl-GL instead exhibited an opposite trend, with significantly higher levels at 18°C than at 12°C under a short-day and non-significant differences under a long-day photoperiod. Generally, total aliphatic GL content, was 33% higher under long-day at 12°C than 18°C, while no-significant difference was found under short-day photoperiod. Regarding indole GLs, the contents of 4-hydroxy-indole-3-yl-methyl-GL and 1-methoxy-indole-3-yl-methyl-GL were greater at 18°C under short-day than under long-day photoperiod, whereas no differences were registered between photoperiods when temperature decreased to 12°C. Generally, total indole GLs were mostly affected by temperature and their content increased by 24% at 18°C with respect to 12°C. The discrepancy in the observed responses may be ascribed to the different optimum preferences for light and temperature of the Gs metabolic enzymes and isoforms (14, 44). It also seems that even the variation between day/night temperatures may also influence the content of GLs in broccoli. Previously, Steindal et al. (93) monitored the contents of individual GLs in broccoli florets grown at different temperatures (15/9 and 21/15°C) and day length (12 and 24 h). The authors revealed that 4-methylsulphinylbuty-GL was unaffected by variations in temperatures (15/9 and 21/15°C). 4-methoxy-3-indolylmethyl-GL and indole-3-yl-methyl-GL were unaffected by day length (12 and 24 h). Generally, high day/night temperatures were associated with peaks in total GLs (31.5–33.2 mg/g dw) regardless of the photoperiod. Under greenhouse conditions at 7–13°C, daily mean temperatures were associated with a radiation of 10–13 mol/m^2^/day; Schonhof et al. (91) noticed an 8-fold increase in methylsulphinylbuty-GL and 3-methylsulphinylpropyl-GL content and a decrease in 3-indolylmethyl-GL.

With regard to seasonal variation, discrepant results and conclusions are drawn, which reflects the complexity of interacting factors shaping the levels of GLs in broccoli. Charron et al. (94) found that 4-methylsulfinylbutyl isothiocyanate content was higher in fall harvests than in spring in broccoli cultivars Brigadier and Emperor. However, studying the effect of seasonal variation on the content of GLs in florets, leaves, and stems of various broccoli cultivars (05-C3, AMaGi, BaeRiDom, CheongJae, Diamond, Grace, Grandeur, JikNok No. 28, NokJae, NokYeom No. 1, TS-2319, and YuDoRI No. 1), Bhandari and Kwak (75) revealed that the highest total GL content was measured in broccoli florets grown in spring (2.26–17.81 μmol/d dw) compared to the fall season (3.17–13.8 μmol/d dw). Conversely, in broccoli stems, higher total GL content was measured in fall (2.91–7.92 μmol/d dw) compared to spring season (1.04–5.74 μmol/d dw). Nevertheless, 4-methylsulfinylbutyl-GL was the main GL in all cultivars regardless of the growing season. Unlike Bhandari and Kwak (75), Pék et al. (95), studying the effect of harvest season (spring/autumn) on 4-methylsulfinylbutyl isothiocyanate concentration of broccoli florets (cultivar Parthenon) under different irrigation regimes and sulfur supply, revealed greater 4-methylsulfinylbutyl isothiocyanate content in fall (32.2–99.9 mg/kg fw) than in spring (9.9–13.3 mg/kg fw) harvests. Differences were also evidenced among first-fall and last-fall harvests, with the latter showing 164–261% higher methylsulfinylbutyl isothiocyanate depending on the applied treatments. This might suggest differences in optimal temperature required for the functional enzymes required for the synthesis of particular GLs in different cropping seasons. According to Verkerk et al. (44), higher levels of total and individual GLs are measured in late (August–January) compared to early (April–July) cropping seasons.

#### Agricultural Practices

Irrigation and fertilization are known to alter the balance between nitrogen and sulfur in the soil, thus presumably altering the content and profile of GLs among other secondary metabolites in plants (86, 96, 119). In addition, controlled pre-harvest salinity stresses have also been applied to improve the yield and health-promoting bioactivity of various fresh products and maintain long shelf-life for minimally processed and processed products (120, 121).

Generally, discrepant conclusions are available regarding the effect of water supply on the GL content in *Brassica*, and it seems that it is species-dependent with implication of other environmental factors. Low rainfall increased the levels of methylsulphinylbuty-GL and methylsulphinylbuty-isothiocyanate in most of the *Brassica* vegetables but decreased their levels in red radish roots. A similar result was also obtained by Radovich et al. (122), who reported that total and individual GLs were also lower in irrigated than non-irrigated cabbage during head development.

Previously, Vallejo et al. (88) found cultivar-dependent response on total GL content of marketable broccoli heads (56 DAT) under rich and poor sulfur fertilization. In cultivars Monterrey and Vencendor, a ~10% decrease and ~69% increase of total GL content was associated with rich sulfur supply, respectively, while GL content in cultivar Marathon remained statistically unchanged. However, Rangkadilok et al. (86) revealed a significant increase (50–70%) in 4-methylsulfinylbutyl-GL content in florets and whole plant fractions (cultivar Marathon) under low (23 kg S/ha) and high (92 kg S/ha) sulfur supply regimes, respectively, throughout maturity stages of the three broccoli cultivars namely Claudia, Marathon, and TB-234. Additionally, the levels of 4-methylsulfinylbutyl-GL content were more responsive to sulfur supply in cultivar Claudia than cultivar Marathon or TB-234. Zaghdoud et al. (96) found considerable decrease (60–80%) in 3-butenyl-GL content with decreasing ${\text{NO}}_{3}^{-}$/${\text{NH}}_{4}^{+}$. However, the levels of indole-3-yl-methyl-GL and 1-methoxy-3-indolylmethyl-GL, as well as total indole GLs, significantly increased with decreasing ${\text{NO}}_{3}^{-}$/${\text{NH}}_{4}^{+}$ ratio and elevated CO_2_ concentrations, suggesting the need for a careful consideration of N-fertilization under projected climatic change for a proper management of broccoli nutritional quality.

Agricultural management is also known to quantitatively and qualitatively affect crop production and the content of secondary metabolites GLs among others. Once again, the effect on GLs is controversial between conventional and organic farming practices. The simple application of olive tree pruning-based biochar increased GL content in broccoli florets with respect to conventionally fertilized treatments, which registered the lowest concentration of 1-methoxy-3-indolylmethyl-GL and methylsulphinylbuty-GL (97). A similar result was also reported by Meyer and Adam (98), where organically grown broccoli exhibited significantly higher amounts of 3-indolylmethyl-GL than their conventionally grown counterparts. However, Vicas et al. (99) noticed 240 and 6% higher 3-indolylmethyl-GL and 1-methoxy-3-indolylmethyl-GL content under conventional compared to organic farming.

Priming and elicitation are strongly recommended eco-friendly agricultural approaches for agricultural production under actual global climate change (123). A number of agents, such as cold, drought, water, inorganic salts, sugars, solid medium with water and various nutrients, beneficial microbes, micronutrients, hormones (JA, MeJA, SA, and ABA), rhizobacteria, as well as organic sources (112, 124–128), are used for fruit and vegetable seed priming. Discrepant conclusions are drawn regarding the effect of NaCl for different broccoli cultivars even when the same treatments are applied. Under moderate salinity stress, Machado and Serralheiro (100) reported an accumulation of GL content and polyphenol contents, leading to an overall improvement of the nutritional quality of broccoli florets. Conversely, López-Berenguer et al. (101) noticed an increase in GL levels in broccoli florets (cultivar Marathon) disregarding the concentrations of NaCl applied (40 or 80 mM). This discrepancy can be ascribed to the date of application of the salt stress and the resistance of the cultivar to such stress. Similarly, Zaghdoud et al. (102) even found an opposite response among two broccoli cultivars (Parthenon and Naxos) to salinity stress (0, 30, 60, and 90 mM NaCl). In fact, while the content of total GL significantly decreased in Naxos due to a sharp decline in the content of indole GLs, it was practically unaffected in Parthenon. Nevertheless, under the applied stress, florets of Naxos accumulate and store higher aliphatic GLs compared to Parthenon, suggesting the possible use of this cultivar with improved health-promoting bioactivity under salinity stress. Similarly, Del Carmen Martínez-Ballesta et al. (43), also using the cultivar Parthenon, found that the content of aliphatic GLs (methylsulphinylbuty-GL and 4-methylthiobutyl-GL) generally increased whereas indole GLs (4-hydroxy-3-indolylmethyl-GL, 3-indolylmethyl-Gl, 4-methoxy-3-indolylmethyl-GL, and 1-methoxy-3-indolylmethyl-GL) decreased in plant grown under salinity stress. However, Sarikamiş and Cakir (103) noticed a decrease in 4-methylsulfinylbutyl-GL and 4- 4-methoxy-3-indolylmethyl-GL in cultivar Marathon by 61 and 74%, respectively, 1 day following 80 mM saline treatment, whereas indole-3-yl-methyl-GL and 3-methylsulfinylpropyl-GL decreased by 65 and 70% respectively, at day 6 compared to the control plants. Generally, except for 4-methoxy-3-indolylmethyl-GL, the authors noticed a decreasing trend for all GLs between 40 and 80 mM followed by a slight increase of all individual GLs. The increase might be due to *de novo* synthesis of GLs once input substrates are available following catabolic reactions affecting other molecules. Moreno et al. (104) pointed out that the combination of salinity and foliar sprayed elicitors (methionine, tryptophan, and chitosan) may increase the content of various secondary metabolites in broccoli florets produced under hydroponic conditions. The effect of salinity stress on GLs seems also to be dependent upon the growth stage of broccoli head. Di Gioia et al. (105) reported an increase in indole GLs (3-indolylmethyl-GL and 1-methoxy-3-indolylmethyl-GL) following irrigation of broccoli florets with saline water (4 dS/m) from transplanting to appearance of the inflorescence. However, total GLs remained unchanged in broccoli heads exposed to the same salinity stress from transplanting to harvest.

Methyl jasmonate and salycilic acid function as signaling molecules; their synthesis is triggered following tissue wounding and aggressor attacks (44), eliciting cascades of reactions leading to the accumulation of various secondary metabolites, such as GLs, among others, as a defensive response. At sprouting stage, Baenas et al. (106, 107) comparatively evaluated the effect of phytohormones (25–250 μM JA or MeJA), sugars (277 mM glucose, 146 mM sucrose), methionine (1–10 mM). and SA (100 μM) in eliciting GL biosynthesis and revealed that most of the applied treatments increased the total GL contents, though differentially, with MeJA resulting the most effective response even at low concentrations. Aliphatic GLs, such as 4-methylsulfinylbutyl-GL and indole GLs, such as indole-3-yl-methyl-GL, 4-methoxy-3-indolylmethyl-GL, and 1-methoxy-3-indolylmethyl-GL levels were, interestingly, increased by 48–247% following 25 μM MeJA treatments, with 4-methylsulfinylbutyl-GL rising from 183 to 294 mg/100 g fw. Following broccoli microgreens treatment with 10 mM CaCl_2_, a significant increase in indole GLs (indole-3-yl-methyl-GL, 1-methoxy-3-indolylmethyl-GL, and 4-hydroxy-3-indolylmethyl-GL) and aliphatic GLs (4-methylthiobutyl-GL, 3-methylsulfinylpropyl-GL, 3-methylthiopropyl-GL, and 4-methylsulfinylbutyl-GL) content was observed, while a low concentration of 1 mM CaCl2 was found ineffective (108, 109). A similar response was observed in broccoli florets by Ku et al. (110) who reported an increase in the biosynthetic activity of some GL degradation products like 4-methylsulfinylbutyl isothiocyanate, neoascorbigen, N-methoxyindole-3-carbinol, and phenethyl isothiocyanate in broccoli florets subjected to pre-harvest application of 500 ppb MeJA and post-harvest treatments with 1-methylcyclopropene assessed in fresh- and in 10–30 days post-harvest stored florets at 4°C, with particular increase in the levels of β-thioglucoside glucohydrolases after application of MeJA (110), whereas Yang et al. (111) noticed a 50% increase in 4-methylthiobutyl-GL as compared to the control in 10 mM CaCl_2_-treated broccoli sprouts. Hassini et al. (112), applying various priming/eliciting agents (K_2_SO_4_ and NaCl solutions, MeJA, SA and methionine), to broccoli plants, reported a 19% increase in total aliphatic GL content after K_2_SO_4_ application, mainly ascribed to a peak in methylsulphinylbuty-GL and 765% increase in total indole GL content following MeJA foliar spray application. However, a 99% decrease in indole-3-yl-methyl-GL levels occurred following all the treatments with the greatest decrease (99%) following KCl priming. A similar trend was also monitored following the pre-harvest treatment of broccoli (Diplomat and Gypsy cultivars) sprouts, young leaves, and florets, with 25 μM sodium selenate (Na_2_SeO_4_), which decreased the levels of amino acids involved in the synthesis of both aliphatic- and indole GLs in broccoli florets, and a 50% decrease in 4-methylsulfinylbutyl-GL and 1-methoxy-3-indolylmethyl-GL were consequently observed (113). This fact may be ascribed to competitive uptake between Se and S in plants.

Under prolonged UV-B exposure, at least four genes involved in GL biosynthesis were downregulated in Arabidopsis, whereas short-term exposure led to enhanced GL content, probably following the activation of jasmonic acid and salicylic acid pathways (129). Pre-harvest and/or supplementary UV radiation might be also beneficial in increasing levels of health beneficial substances and altering the pathways related to the biosynthesis of various secondary metabolites, particularly nitrogen-containing molecules (115, 130, 131). The pre-harvest treatment of broccoli sprouts treated with UV-B radiation at doses ranging from 0.3 to 1 kJ/m^2^/d prompted an increase in the levels of both aliphatic and indole GLs (114). Using a combined treatment (10 mM CaCl_2_ and 0.18 Wh/m^2^ UV-B) on young broccoli microgreens (shoots of the young plant), an increase in GL content correlated essentially with an increase in singrin content at 8.8 kJ/m^2^/d was obtained (115).

### Post-harvest Factors Affecting GL Content

Generally, fresh is used for freshly harvested products. However, those sold in various markets and shops were already prone to various post-harvest factors at different levels. Several post-harvest factors have significant effect in altering broccoli GLs in terms of quality and quantity. These include the storage, processing, and packaging. An overview of the current state of research and of the key results is given in Table 2.

**Table 2:** The effect of post-harvest manipulations on the level of broccoli glucosinolates (GL class, individual GLs, and/or GL biosynthetic enzymes).

**Post-harvest manipulations**	**Targeted GLS class/Individual GLS/GLS Biosynthetic enzymes**	**Cultivar name and/or particular specifications**	**Effect on GLS class/Individual GLS/GLS Biosynthetic enzymes**	**References**
**STORAGE**				
48 h storage at room temperature	4-Hydroxy-3-indolylmethy-GL 4-Methoxy-3-indolylmethyl-GL	Cv. 1997	4-hydroxy-3-indolylmethy-GL and 4-methoxy-3-indolylmethyl-GL increased in 48 h stored broccoli heads at room temperature	(132)
Controlled atmosphere storage at 5, 10, and 18°C			4-hydroxy-3-indolylmethy-GL and 4-methoxy-3-indolylmethyl-GL increased in broccoli florets stored under controlled atmosphere at 3 different temperatures (5, 10, and 18°C)	
6-days storage until 12 days at 0, 5, and 10°C	4-Methylsulfinylbutyl-GL	Cv. Luling	4-methylsulfinylbutyl-GL content increased in broccoli florets after 6-days storage followed by a decreasing trend regardless temperature (0, 5, and 10°C) 4-methylsulfinylbutyl-GL content can be preserved until 12 days storage under 0–5°C	(133)
Storage of broccoli heads for 9 days at 1–2°C and 85–90% RH	1-Methoxy-3-indolylmethyl-GL	NA	1-methoxy-3-indolylmethyl-GL increased 10-fold	(134)
6-benzylaminopurine (6-BA) floret treatment	4-Methylsulfinylbutyl-GL isothiocyanate	Cv. Chaoda No. 1	4-methylsulfinylbutyl-GL content increase	(135)
500 μL/L ethanol floret treatment				(136)
Pre-harvest MeJA treatment of broccoli florets	4-Methylsulphinylbutyl-GL 2-Phenylethyl-GL	Cv. Green Magic	Increase in 4-methylsulphinylbutyl-GL and 2-phenylethyl-GL after pre-harvest MeJA	(110)
1-MCP application to 10–30 days-stored broccoli florets at 4°C			3-indolylmethyl-GL content unchanged	
Storage of broccoli heads at 20°C for 24 h	4-Hydroxy-3-indolylmethyl-GL	NA	4-hydroxy-3-indolylmethyl-GL content increased by 84% increase	(137)
Exogenous ethylene (1,000 ppm)treatment of broccoli florets for 24 h	4-Methylsulfinylbutyl-GL 4-Hydroxy-3-indolylmethyl		4-methylsulfinylbutyl-GL and 4-hydroxy-3-indolylmethyl increased by ~52 and ~223%, respectively compared to fresh samples	
Ethylene or MeJA treatment of wounded broccoli heads	1-Methoxy-3-indolylmethyl-GL		193 and 286% increase in the levels of 1-methoxy-3-indolylmethyl-GL, respectively	
Application of melatonin to broccoli florets at concentration ranging from 1–100 μM	4-Methylsulfinylbutyl-GL 4-Methylsulfinylbutyl isothiocyanate	NA	Significant increase in 4-methylsulfinylbutyl-GL and 4-methylsulfinylbutyl isothiocyanate contents	(138)
	GLs biosynthetic genes Elong, CYP83A1, MYB28, UGT74B1 and FMOGS-OX1 and AOP2		Up-regulation of *Elong, CYP83A1, MYB28, UGT74B1*, and *FMOGS-OX1* and down-regulation of *AOP2*	
1 week pre-storage at 1°C and 3-days storage at 15°C of broccoli florets	Total GLs	Cv. Marathon	80% decrease in total GL content	(139)
Storage of 1-methylcyclopropene treated broccoli florets for 5 days at 20°C	Total GLs	Cv. Lvxiong	Considerable decrease in total GLs	(140)
Sucrose treatment of broccoli florets throughout 4-days storage at 20°C and 95% RH	Total GLs 4-Methylsulfinylbutyl isothiocyanate	Cv. Youxiu	GLs and 4-methylsulfinylbutyl isothiocyanate contents levels are maintained unchanged	(141)
Broccoli heads storage for 9 days at 1–2°C and 85–90%	Indole-3-yl-methyl-GL 4-Methoxy-3-indolylmethyl-GL 1-Methoxy-3-indolylmethyl-GL	NA	50% decrease in indole-3-yl-methyl-GL and 4-methoxy-3-indolylmethyl-GL and up to 10-fold increase in the level of 1-methoxy-3-indolylmethyl-GL	(134)
Cold storage of broccoli florets for 5 days at 4°C, for 7 days at 4–8°C or for 2 days at 25°C with subsequent exposure to fluorescent light	Total GLs	Cv. Tokyo Dome	Total GLs remained unchanged or little affected	(142)
		NA		(143)
		Cv. Parthenon		(144)
		Cv. Chaoda No. 1		(145)
Storage of broccoli heads at temperatures of 1 or 4°C, 99% RH, for 2, 7, 14, or 28 days followed by exposure to 8, 15, or 20°C, with 99, 90, or 70% RH, respectively, for 3 days	4-Methylsulfinylbutyl-GL	Cv. Marathon	4-methylsulfinylbutyl-GL content remained unchanged	(146)
Pre-storage of broccoli florets at 0 or 4°C for 4 or 7 days	Total GLs	Cv. Marathon	No significant changes in the contents of total GLs	(82)
Broccoli sprouts storage for 2 weeks at 4°C and 95% RH and treated with fennel, caraway, basil, thyme, and sage essential oils	Total GLs	Cv. Sakura	The antioxidant content of broccoli sprouts increases 2.18% loss in GLs compared to more than 49% in non-treated sprout samples in thyme essential oil-treated sprouts	(147)
Treatment of microgreens with 1 mM CaCl_2_ spray and UV-B and storage 21 days	4-Methylthiobutyl-GL	Cv. De Cicco	57% decrease in 4-methylthiobutyl-GL content	(109)
Treatment of microgreens with CaCl_2_ concentration of 10 mM and storage 21 days	Aliphatic GLs		Increase in aliphatic GLs and 35–43% decrease in GLs degradation rate	
Exposure of florets to 7-days pre-storage at 0°C followed by 3-days storage at 10°C under visible light of 13 μmol/m^2^/s and 25 μmol/m^2^/s	3-Methylsulfinylpropyl-GL 4-Methylsulfinylbutyl-GL Total aliphatic GLs	Cv. Marathon	3-methylsulfinylpropyl-GL, 4-methylsulfinylbutyl-GL and total aliphatic GL content increased, respectively by 130, 99, and 102%	(82)
Exposure of florets to 7-days pre-storage at 0°C followed by 3-days storage at 18°C under visible light of 25 and 25 μmol/m^2^/s	4-Hydroxy-3-indolylmethyl-GL		4-hydroxy-3-indolylmethyl-GL increased considerably	
UV-B irradiation of broccoli florets using intensities ranging from 0 to 5 W/m^2^ and doses ranging from 0 to 12 kJ/m^2^ throughout 17-days storage at 4°C	4-Methylsulfinylbutyl-GL	Cv. Legacy	Accumulation of 4-methylsulfinylbutyl-GL content until 18 h after treatment	(148)
Effect of UV, UV-B treatments or their combination on florets subjected to 4–7 days dark pre-storage, subsequent 10°C 3-days storage and left in the dark	Total GLs		Total GL content for remains unchanged	
**PROCESSING AND MINIMAL PROCESSING**				
Postharvest physical injuries of plant tissue after grinding, slicing, mixing, juicing, cooking, and frosting/defrosting	Isothiocyanates	NA	Accumulation of isothiocyanates and GLs degradation products	(149)
		NA		(150)
		NA		(151)
		NA		(152)
Cutting of broccoli heads into florets and subsequent incubation for 24 h at 20°C	Total GLs	NA	490% increase in total GLs content	(150)
	4-Hydroxy-3-indolylmethyl-GL		4,200% increase in 4-hydroxy-3-indolylmethyl-GL content	
	1-Methoxy-3-indolylmethyl-GL		1,300% increase in 1-methoxy-3-indolylmethyl-GL contents	
Chopping and storage of broccoli florets	4-Hydroxy- and 4-methoxy-3-indolylmethyl-GL	NA	3.5- and 2-fold increase in 4-hydroxy- and 4-methoxy-3-indolylmethyl-GL content, respectively and significant decline of most GLs	(44, 153)
Combined treatment with 250 ppm MeJA and ultrasound (20 min, frequency 24 kHz, amplitude 100 μm) after 3-days storage at 15°C	4-Hydroxy-3-indolylmethyl- G	Cv. Tlaloc	187% increase in 4-Hydroxy-3-indolylmethyl-GL content	(154)
	4-Methylthiobutyl-GL		112% increase in 4-methylthiobutyl-GL content	
	2-Phenylethyl-GL		755% increase in 2-Phenylethyl-GL content	
	1-Methoxy-3-indolylmethyl-GL		232% increase in 1-Methoxy-3-indolylmethyl-GL content	
Pulsed-light treatment (1.5 kJ/m2 UV-C), water-washing or chlorine-sanitizing of fresh-cut broccoli florets	2-Phenylethyl-GL	NA	2-Phenylethyl- and 2-Phenylethyl-, 4-methylsulfinylbutyl-, and 2(R)-2-Hydroxy-3-butenyl-GLs contents significantly increased after 24 h	(151)
	2-Phenylethyl-GL			
	4-Methylsulfinylbutyl-GL			
	2(R)-2-Hydroxy-3-butenyl-GL			
3 UV-C light pulses of 1.5 kJ/m^2^ (24 h)	4-Methylsulphinylbutyl	Bimi broccoli	132% increase in 4-Methylsulphinylbutyl-GL content	(83)
	4-Methylsulphinylbutyl isothiocyanate		122% in 4-Methylsulphinylbutyl isothiocyanate content	
	2-Phenylethyl-GL		50% decrease in 2-Phenylethyl-GL content after 24 h	
Single treatment using UV-B (15 kJ/m^2^) and a combination of UV-B (15 kJ/m^2^) + UV-C (9 kJ/m^2^) induced	4-Methylsulphinylbutyl-GL		The highest 4-methylsulphinylbutyl-GL content (131 and 117 mg kg^−1^) in Bimi broccoli florets after 72 h	
UV-B treatment	Total GLs	NA	Up-regulation of GL biosynthesis	(114)
High pressure processing (100–600 MPa) of 6-days-old broccoli sprouts	GLs-myrosinase system and isothiocyanate production	NA	Beneficial effect on GLs-myrosinase system with a conversion rate of GLs to isothiocyanate attaining 18% until 300 MPa and 85% upward	(152)
Thermal treatment of broccoli florets at 45 and 70°C	Myrosinase	NA	Remains active until 45°C Loose more than 90% of the activity after 10 min at 70°C	(155)
15 min high pressure processing (200–500 MPa) at 40°C and after 35 min using 300 MPa.	4-Methylsulphinylbuty-GL	NA	The highest conversion rate of methylsulphinylbuty-GL to methylsulphinylbuty-isothiocyanate in broccoli florets was attained after	(155)
	4-Methylsulphinylbuty-isothiocyanate			
High pressure processing (600 MPa) at room temperature or at 75°C	4-Methylsulphinylbutyl-isothioyanate	NA	Remains unchanged	(156)
Ordinary and high pressure cooking	Total GLs Indole- and aliphatic GLs	Cv. Marathon	33–55% decrease in total GL content after both treatment affecting mostly the indole than aliphatic GLs	(157)
Low pressure steaming, boiling, and sous vide processing	Total GLs	Kailan-hybrid broccoli Cv. Bimi	40–80% decrease in total GLs content	(158)
Pasteurization Freezing	Isothiocyanates	NA	Significant decrease in isothiocyanates content Unchanged levels after freezing	(159)
Broccoli hummus microwaving (9 kW/40 s) or high-pressure processing (550 MPa/10 min/23°C) and storage until 4 weeks at 5°Cs	Methylsulphinylbuty-GL	Cvs Parthenon and Bimi	110% higher methylsulphinylbuty-GL content after high pressure processing	(160)
	Methylsulphinylbuty-isothiocyanate		Methylsulphinylbuty-isothiocyanate content was better maintained until 4 weeks at 5°C after microwaving	
Exposure to 4 kV/cm for 525 and 1,000 μs	Total GLs	Cv. Parthenon	187–212% increase in GL content of broccoli florets and 111–203% in stalks	(161)
Pulsed electric field treatment of broccoli purée and juice	β-thioglucoside glucohydrolases activity	NA	Increasing enzymatic activity and degradation of most GLs	(162)
Cyclodextrins application	4-Methylsulfinylbutyl-GL	NA	Cyclodextrins stops the decrease affecting 4-methylsulfinylbutyl-GL content in broccoli juice	(163)
Mild heat treatment	Aliphatic and indolic GLs	NA	Aliphatic GL content is unaffected 90% decrease in indolic GLs content	(164)
Blanching Cooking	Total GLs	Kailan-hybrid broccoli Cv. Bimi	27–30% loss after blanching 72% loss after cooking	(158)
		Cv. Sebastian		(165)
		NA		(19)
		NA		(166)
Steaming for 1–3 min Blanching Microwaving Heating Boiling	4-Methylsulfinylbutyl isothiocyanate	Cvs Aviso, Dania, Grafitti, Emeraude, and Celio	4-methylsulfinylbutyl isothiocyanate content was better preserved after steaming for 1–3 min than blanching, microwaving, heating,– or boiling	(167)
		Cvs Pinnacle, Marathon, and Patriot		(77)
		NA		(168)
		NA		(169)
		NA		(166)
		NA		(45)
Boiling of green and purple cauliflower for 15 min at 100°C	Total GLs and Isothiocyanates	Cv. Graffiti	Significant decrease in total GL content and their degradation products	(170)
Heat treatment of broccoli florets at 41°C for 180 min or 47°C for 12 min	Indole GLs	Cv. Diplomat	Significant increase in indole GL content	(171)
Heat treatment of broccoli florets at 60°C	Total GLs	NA	Significant loss in total GL content	(134)
Heat treatment of different *Brassica* crops	3-Indolylmethyl-GL 4-Methoxy-3-indolylmethyl	NA	3-indolylmethyl-GL and 4-methoxy-3-indolylmethyl were most stable in red cabbage compared to broccoli, Brussels sprouts, pak choi, and Chinese cabbage	(172)
	3-Butenyl-GL		3-butenyl-GL content was most stable in broccoli after 120 min at 100°C	
Binary processing: broccoli florets/onion	Total GLs	NA	Higher preservation of GL content compared to single florets processing	(173)
Sous-vide boiling of broccoli florets	Total GLs	Kailan-hybrid broccoli Cv. Bimi	80% loss in GL content of new kailan-hybrid broccoli.	(158)
High temperature (100–130°C) processing	Aliphatic and indolic GLs	Cv. Calabrese	Hydroxylated GLs are less stable than aliphatic	(174)
**PACKAGING**				
Modified Atmosphere Packaging	Aliphatic, indolic and aromatic GLs	Cv. Parthenon	Polyethylene-, polypropylene- and polystyrene-packaging better preserved florets GL content until 3 weeks even at relatively higher temperatures	(144)
		Cv. Legacy		(175)
		Cv. Aishwarya		(176)
		Cv. Lord F1		(177)
Innovative modified atmosphere packaging (8.2% CO_2_ and 2.0% O_2_)	Total GLs	NA	Antioxidant composition of broccoli florets maintained up to 25-days' storage	(178)
Controlled atmosphere packaging (0.5% O_2_ + 20% CO_2_)	Total GLs	Cv. Marathon	21–42% increase in total GL content, respectively after storage under controlled atmosphere and air with respect to fresh floret	(179)
	3-Indolylmethyl-GL		3-indolylmethyl-GL content levels declined by 35% in broccoli stored 7 days at 10°C under	
	4-Methoxy-3-indolylmethyl-GL		4-methoxy-3-indolylmethyl-GL levels increased	
Modified atmosphere packaging (1% O_2_ +21% CO_2_ and 8% O_2_ + 14% CO_2_ at 8°C)	Aliphatic GLs	Cv. Milady	Aliphatic GL content was preserved in cauliflower florets but decreased in broccoli florets	(180)
Modified atmosphere packaging (8% O_2_ + 14% CO_2_)		Cv. Milady Mini broccoli	Aliphatic and indole GL content is preserved for 7 days	(181)
Modified atmosphere packaging (perforated polyethylene bags)	Aliphatic and indole GLs	Cv. Youxiu	Aliphatic and indole GL content is maintained in broccoli florets until 13 days at 4°C relative to unwrapped florets stored in open boxes	(182)

#### Storage

Broccoli is a highly perishable crop due to its high respiration rate. This results in important economic losses and poses a challenge to post-harvest storage, logistics, and supply management. Therefore, storage conditions play a fundamental role in slowing down fresh product decay and preserving quality. Contrasting reports regarding the effect of storage on GLs are available and it seems that the effect of storage conditions on GL content differs among heads and florets. An increase in various GLS was observed in broccoli florets and heads stored under different conditions. Increased levels of 4-hydroxy-3-indolylmethy-GL and 4-methoxy-3-indolylmethyl-GL were detected in 48 h stored broccoli heads at room temperature and in broccoli (cultivar 1997) florets stored under controlled atmosphere at three different temperatures (5, 10, and 18°C) (132). 4-methylsulfinylbutyl-GL content increased in broccoli florets after 6-days storage followed by a decreasing trend regardless of temperature (0, 5, and 10°C), and their content can be preserved for 12 days' storage under 0–5°C (133). Similarly, Baenas et al. (134) monitored a 10-fold increase in the levels of 1-methoxy-3-indolylmethyl-GL in broccoli heads stored for 9 days at 1–2°C and 85–90% RH.

The content of 4-methylsulfinylbutyl-GL isothiocyanate exhibited a post-harvest increase in 6-benzylaminopurine (6-BA) treated (135) and in 500 μL/L ethanol treated (136) broccoli florets, suggesting the advantageous application of such procedures for double purpose of extending shelf-life while increasing the nutritional quality of broccoli florets. Ku et al. (110) reported also an increase in 4-methylsulphinylbutyl-GL and 2-Phenylethyl-GL after pre-harvest MeJA and a preservation of 3-indolylmethyl-GL content after 1-methylcyclopropene (1-MCP) application to 10–30 days-stored broccoli florets at 4°C, which suggests the useful application of those treatments in controlling the nutritional quality of stored broccoli. 4-hydroxy-3-indolylmethyl-GL content exhibited an 84% increase when whole local market broccoli heads were stored at 20°C for 24 h, but the application of exogenous ethylene (1,000 ppm) for 24 h increased the concentration of 4-methylsulfinylbutyl-GL and 4-hydroxy-3-indolylmethyl by ~52 and ~223%, respectively, compared to freshly harvested samples (137). Also, considerable increase in the levels of 1-methoxy-3-indolylmethyl-GL, attaining 193 and 286%, respectively, was detected in wounded heads in response to ethylene or MeJA treatments. This is mainly explained by the activation of *CYP81F* genes transcription producing methoxylated derivatives of indole-3-yl-methyl-GL and hydroxylated derivatives (4-hydroxy-3-indolylmethyl-GL) following ethylene and MeJA application (137).

The use of melatonin (N-acetyl-5-methoxytryptamine) in agriculture has increased particularly since it protects plant from various biotic and abiotic stresses by modulating key physiological process (183, 184). The application of melatonin to broccoli florets at concentrations ranging from 1 to 100 μM has been associated with significant increase in 4-methylsulfinylbutyl-GL and 4-methylsulfinylbutyl isothiocyanate contents and was also detected with synchronized upregulation of various GL biosynthetic genes, such as *Elong, CYP83A1, MYB28, UGT74B1*, and *FMOGS*-*OX1*, and downregulation of *AOP2*. Miao et al. (138) noted the additional activation of *BoTGG1* expression in broccoli florets treated with 1 μM melatonin and stored at room temperature.

Under other particular conditions, a decrease affecting different classes of GLs and individual GLs was also reported. Vallejo et al. (139) noticed up to an 80% decrease in total GL content of broccoli florets (cultivar Marathon) subjected to 1 week of pre-storage at 1°C and 3 days' storage at 15°C. Similarly, Yuan et al. (140) found a considerable decrease in total GLs in 1-methylcyclopropene-treated broccoli florets and stored for 5 days at 20°C. Xu et al. (141) reported that the decrease in GLs and 4-methylsulfinylbutyl isothiocyanate contents of broccoli (cultivar Youxiu) florets were suppressed by sucrose treatment throughout the 4-days storage period at 20°C and 95% RH. Similarly, Baenas et al. (134) noticed a 50% decrease in indole-3-yl-methyl-GL and 4-methoxy-3-indolylmethyl-GL, with concomitant increase (up to 10-fold) in the level of 1-methoxy-3-indolylmethyl-GL throughout 9 days' storage of broccoli heads stored at 1–2°C and HR of 85–90%.

In other experiments, the content of GLs remained unchanged or little affected, such as cold storage of broccoli florets for 5 days at 4°C, for 7 days at 4–8°C, or for 2 days at 25°C with subsequent exposure to fluorescent light (142–145). Similarly, 4-methylsulfinylbutyl-GL content remained unchanged in broccoli (cultivar Marathon) heads stored at temperatures of 1 or 4°C, 99% relative humidity (RH), for 2, 7, 14, or 28 days, followed by exposure to 8, 15, or 20°C, with 99, 90, or 70% RH, respectively, for 3 days (146). No significant changes in the contents of total GLs were found during pre-storage of broccoli florets (cultivar Marathon) at 0 or 4°C for 4 or 7 days (82). Therefore, the β-thioglucoside glucohydrolases' hydrolytic activity and the GLs' biosynthetic activity are key forces driving the GL level of stored broccoli florets (140, 182). The proper use and application of different post-harvest treatments might ensure a better control of GL content and fresh produce with high nutritional value and beneficial health effects. Treatment with essential oils is also a promising eco-friendly practice to improve the storability and conservation of many horticultural crops (185). El-Awady et al. (147) evaluated the feasibility in using fennel, caraway, basil, thyme, and sage essential oils to preserve the horticultural traits and the GL levels in broccoli sprouts during storage at 4°C and 95% RH. The authors noticed an increase in antioxidant content of broccoli sprouts following the application of all tested essential oils compared to the control. In thyme essential oil-treated sprouts, there was 2.18% loss in GLs compared to more than 49% in non-treated sprout samples at the end of the storage period (2 weeks).

Post-harvest light treatments of broccoli sprouts and florets under storage were reported to trigger changes affecting GL biosynthesis (109, 186). At early stages, it also seems that the beneficial effect of UV-B treatment is dependent upon CaCl_2_ concentration when combined. There was a decrease (57%) in 4-methylthiobutyl-GL content after the treatment of microgreens with 1 mM CaCl_2_ spray and UV-B. However, when microgreens were treated by higher CaCl_2_ concentration of 10 mM, an increase in aliphatic GLs and 35–43% decrease in GL degradation rate throughout 21 days was noticed regardless of the applied light treatment (109). In mature florets, 3-methylsulfinylpropyl-GL, 4-methylsulfinylbutyl-GL, and total aliphatic GL content increased, respectively by 130, 99, and 102% after exposure to 7 days' pre-storage at 0°C followed by 3 days' storage at 10°C under visible light of 13 and 25 μmol/m^2^/s, compared to freshly harvested samples. However, under higher visible light intensity (25 μmol/m^2^/s) and higher storage temperature (18°C), 4-hydroxy-3-indolylmethyl-GL increased considerably in broccoli heads exposed to a 7-days pre-storage period at 0°C and 3-days' storage at 10°C (82). Darré et al. (148) reported differential response of GLs according to the applied treatment, storage condition, and duration while assessing the effect of various UV-B irradiation intensities (low to high) ranging from 0 to 5 W/m^2^ and doses ranging from 0 to 12 kJ/m^2^ on the overall nutritional quality of broccoli florets throughout 17 days of storage at 4°C. The short-term response was an accumulation of 4-methylsulfinylbutyl-GL in broccoli florets until 18 h after UV-B treatment. However, UV treatments, UV-B treatments, or their combination was ineffective in altering total GLs for samples subjected to 4–7 days' dark pre-storage, subsequent 10°C 3-days storage, and left in the dark.

#### Processing and Minimal Processing

Before being consumed, vegetables crops of the *Brassica* groups are jeopardized by different home and industrial processing. It has been reported that treatments, such as slicing, cooking, steaming, frying, and microwaving significantly affect the levels of GL content in broccoli. Naturally, postharvest physical injuries of plant tissue after grinding, slicing, mixing, juicing, cooking, and frosting/defrosting prompt an activation of the myrosinase-GL system, which leads to the accumulation of isothiocyanates and GLs degradation products (149–152). Torres-Contreras et al. (150) reported that cutting of broccoli heads into florets and subsequent incubation for 24 h at 20°C triggered an increase attaining 490% in total GLs, 4,200% in 4-hydroxy-3-indolylmethyl-GL, and 1,300% in 1-methoxy-3-indolylmethyl-GL contents. Similarly, Verkerk et al. (44, 153) observed a 3.5- and 2-fold increase in 4-hydroxy- and 4-methoxy-3-indolylmethyl-GL, respectively, after chopping and storage of broccoli florets, but a significant decline of most GLs. This increase was reported as wound-induced *de novo* GL biosynthesis triggered by ethylene (4-hydroxy-3-indolylmethyl-GL and 1-methoxy-3-indolylmethyl-GL) and by ROS (4-hydroxy-3-indolylmethyl-GL, indole-3-yl-methyl-GL, and 1-methoxy-3-indolylmethyl-GL) (150).

The use of ultrasound leads to microorganisms' and enzymes' inactivation, but might also damage plant tissues if applied at high frequencies and trigger a stress response accordingly. Aguilar-Camacho et al. (154) reported an increase of 187% in 4-hydroxy-3-indolylmethyl-, 112% in 4-methylthiobutyl-, 755% in 2-phenylethyl-, and 232% in 1-methoxy-3-indolylmethyl-GL by a combined treatment with 250 ppm MeJA and ultrasound treatment (20 min, frequency 24 kHz, amplitude 100 μm) after 3 days' storage at 15°C. Collazo et al. (151) noted that 2-phenylethyl- and 2-phenylethyl-, 4-methylsulfinylbutyl-, and 2(R)-2-hydroxy-3-butenyl-GLs were significantly increased 24 h after pulsed-light treatment (1.5 kJ/m^2^ UV-C), water-washing, or chlorine-sanitizing of fresh-cut broccoli florets. The exposure of fresh-cut broccoli to three UV-C light pulses of 1.5 kJ/m^2^ revealed a 132% increase in 4-methylsulphinylbutyl-GL and 122% in 4-methylsulphinylbutyl isothiocyanate but almost 50% decrease in 2-phenylethyl-GL contents after 24 h. A single treatment using UV-B (15 kJ/m^2^) and a combination of UV-B (15 kJ/m^2^) + UV-C (9 kJ/m^2^) induced the highest 4-methylsulphinylbutyl-GL content (131 and 117 mg kg^−1^) in Bimi broccoli florets after 72 h (83). It is widely recognized that UV-B treatment upregulates the biosynthesis of GLs (114).

The interest in non-thermal food processing, such as high-pressure processing, by consumers is increasing due to an increasing concern about the beneficial effect of natural antioxidants generally found in fresh produce. The proper application of this technology helps inactivate food pathogens at ambient temperature, preserving nutritional quality and therefore the shelf-life of the final product. Inconsistent conclusions are also drawn regarding the effect of high-pressure processing on GL content. Using a high pressure in the range of 100–600 MPa, Westphal et al. (152) noticed their beneficial effect on sprouts' GLs-myrosinase system, with a conversion rate of GLs to isothiocyanate attaining 18% until 300 MPa and 85% upwards. It has been reported that broccoli myrosinase remains active until 45°C but loses more than 90% activity after 10 min at 70°C (155). Van Eylen et al. (155) reported that the highest conversion rate of 4-methylsulphinylbuty-GL to methylsulphinylbuty-isothiocyanate in broccoli florets was attained after 15 min using a pressure of 200–500 MPa at 40°C and after 35 min using 300 MPa. However, the conversion rate significantly decreased, most likely due to enzymatic inactivation of myrosinase. Besides, it has been reported that 4-methylsulphinylbutyl-isothioyanate in vegetal matrix remains stable at room temperature or at 75°C and up to 600 MPa pressure (156). However, Vallejo et al. (157), comparing ordinary and high-pressure cooking, reported significant (33–55%) decrease in total GLs after both treatments, affecting mostly the indole rather than the aliphatic class. This may be attributed to cooking temperature rather than pressure intensity. Martínez-Hernández et al. (158) found a 40–80% decrease in total GLs after low-pressure steaming as well as boiling and sous vide of a new kailan-hybrid broccoli. Tríska et al. (159) observed a significant decrease in isothiocyanates content of different cruciferous vegetables-based juices with pasteurization. However, their level remained practically unchanged after freezing. In an innovative broccoli (cultivars Parthenon and Bimi) hummus prepared and subjected to microwaving of 9 kW/40 s or high-pressure processing of 550 MPa/10 min/23°C, Klug et al. (160) noticed 110% higher 4-methylsulphinylbuty-GL after high-pressure processing of Parthenon-based hummus, while 4-methylsulphinylbutyl-isothiocyanate was better maintained until 4 weeks at 5°C after microwaving.

Pulsed electric fields (PEF) have been also proposed as a non-thermal food-grade technology to improve bioactive compounds biosynthesis and reduce microbial load without deleterious effect on the processed product (161, 187, 188). An increase of 187–212% in GL content in broccoli florets and 111–203% in stalks was reported after exposure to 4 kV/cm for 525 and 1,000 μs (161). However, Frandsen et al. (162) noted an increasing enzymatic activity of β-thioglucoside glucohydrolases after PEF treatment of broccoli purée and juice, leading to the degradation of most GLs. The decrease affecting 4-methylsulfinylbutyl-GL attained 71% 24 after juicing, which stresses the need for the application of stabilization agents, such as cyclodextrins to encapsulate valuable compounds in broccoli juice (163).

Thermal processing induces changes in cell-wall structural components, nutrients (including proteins and vitamins), and non-nutritive bioactive components, such as GLs and polyphenols. GLs are relatively stable under heat treatment and their hydrolysis is mainly enzymatic. Nevertheless, it should be highlighted that GL degradation products are responsible for their bioactivity. It seems that the effect of heat treatment on GL content is dependent upon genotype, food matrix, the chemical class of GL, the temperature, and the duration of the treatment (166, 189). At mild heat treatment, aliphatic GLs are unaffected but indole GLs decreased considerably (90%) (164, 190). The loss in total GLs was 27–30% after blanching and increasing to 72% after cooking with a complete inhibition of β-thioglucoside glucohydrolases activity (19, 158, 165, 166). It has been noted that 4-methylsulfinylbutyl-isothiocyanate and the nutritional value of broccoli florets was better preserved after steaming for 1–3 min than after blanching, microwaving, heating, or boiling (45, 77, 166–169). Similarly, boiling of green and purple cauliflower for 15 min at 100°C significantly reduced not only GLs, but also their degradation products (170). In other experiments, treatment at 41°C for 180 min or 47°C for 12 min significantly increased indole GLs in processed broccoli (cultivar Diplomat) florets (171), most likely due to an ROS-induced accumulation triggered by heat stress. At a higher temperature of 60°C a significant loss in total GLs occurred (134). The effect of temperature and other factors on GL content in broccoli is complex since other food matrixes present might also influence the final content of GLs during processing. The effect of temperature on GL content is also dependent on the type of vegetal matrix. In fact, in different pak choi cultivars, boiling for 15 min, reduced the GL content by 54–82.2% with respect to control, while their content remained unchanged after steaming. Dekker et al. (172) also reported differential thermal stability of the same GLs in different *Brassica* crops consisting of red cabbage, broccoli and Brussels sprouts, pak choi, and Chinese cabbage. 3-indolylmethyl-GL and 4-methoxy-3-indolylmethyl were most stable in red cabbage, while 3-butenyl-GL was most stable in broccoli after 120 min at 100°C.

Giambanelli et al. (173) reported a positive and protective interaction of onion with broccoli florets at boiling temperature leading to significantly higher preservation of GLs in a processed broccoli/onion system than in broccoli, suggesting the possible use of such systems in improving the health-promoting bioactivity of processed broccoli products. Under sous-vide boiling, Martínez-Hernández et al. (158) found an 80% loss in GL content of new kailan-hybrid broccoli. Generally, hydroxylated GLs are less stable than aliphatic under high temperature (100–130°C) and more degraded in basic medium than neutral and slightly acidic medium (174).

#### Packaging

Controlled atmosphere storage and modified atmosphere packaging are also a practical alternative for preserving *Brassica* vegetables' quality and marketability during a longer shelf-life (44, 191) with respect to ordinary canning leading to considerable loss of GLs following their thermal degradation (44). It has been reported that polyethylene-, polypropylene-, and polystyrene-MAP better preserved aliphatic, indole, and aromatic GLs as well as the nutritional quality of broccoli florets of various cultivars (Parthenon, Aishwarya, and Lord F1) stored until 3 weeks, even at relatively higher temperatures (144, 175–177). Similarly, acceptable sensory quality texture and antioxidant composition of broccoli florets was maintained up to 25 days' storage after using an innovative MAP made of gas promoter-gas barrier blending materials, maintaining an internal atmosphere of 8.2% CO_2_ and 2.0% O_2_ (178). Hansen et al. (179) monitored a 21–42% increase in total GLs, respectively, after storage under controlled atmosphere of 0.5% O_2_ + 20% CO_2_ and air with respect to fresh florets. Individual GLs, such as 3-indolylmethyl-GL, levels declined by 35% in broccoli stored 7 days at 10°C under 0.5% O_2_ + 20% CO_2_ or 20% CO_2_, whereas 4-methoxy-3-indolylmethyl-GL levels increased during storage under low O_2_ atmosphere and increased further after transfer to air. Rangkadilok et al. (192) measured a 55–56% decrease in 4-methylsulphinylbuty-GL in broccoli stored in open boxes after 3 days and plastic bags after 7 days at 20°C, respectively. However, modified atmosphere packaging at 4°C preserved the 4-methylsulphinylbuty-GL level practically unchanged for up to 10 days with respect to air control packaging. Dissimilar dynamic changes in GL content of 7-days stored broccoli and cauliflower mix under controlled atmosphere packaging has been also reported by Schreiner et al. (180). Aliphatic GLs were preserved in cauliflower florets but decreased in broccoli florets under 1% O_2_ + 21% CO_2_ and 8% O_2_ + 14% CO_2_ at 8°C. Similarly, Schreiner et al. (181) reported discrepant response to storage under modified atmosphere between mini broccoli and mini cauliflower since aliphatic and indole GLs were better preserved under 8% O_2_ + 14% CO_2_ in mini broccoli for 7 days but at 1% O_2_ + 21% CO_2_ in mini cauliflower for 7 days, which highlights the suitable separate packaging of such agricultural crops to avoid the decrease in health-promoting bioactivity. Modified atmosphere packaging treatments maintained the levels of individual aliphatic and indole GLs in broccoli florets relative to those unwrapped florets stored in open boxes, and longer shelf-life with improved GL content was better achieved using less perforated polyethylene bags and lower temperature (13 days at 4°C) (182).

## Conclusions

**Figure 8:**
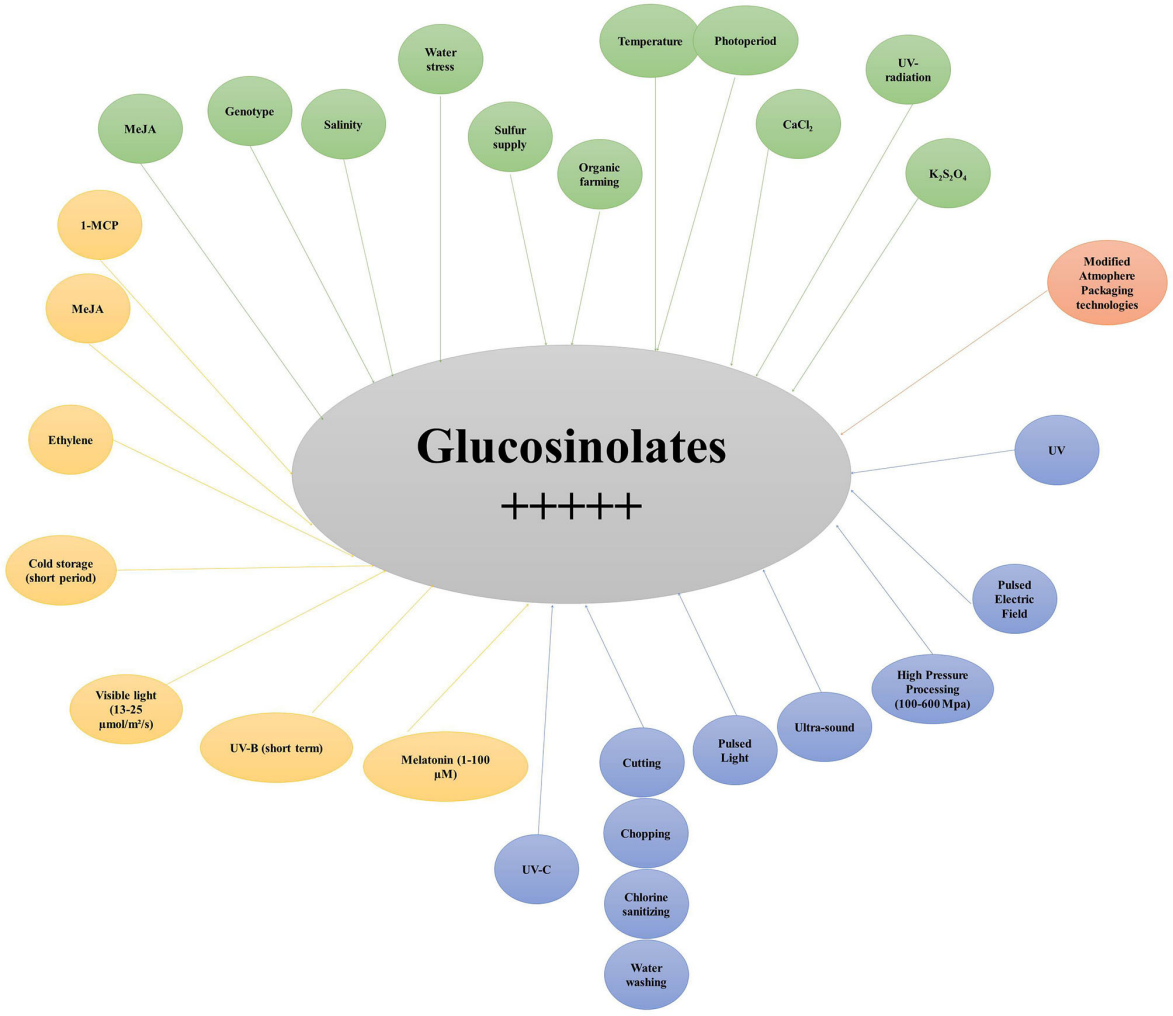
Major pre- and post-harvest factors positively affecting glucosinolate content in broccoli. MeJA, Methyl Jasmonate; 1-MCP, 1-Methylcyclopropene; UV, ultraviolet; UV-B, ultraviolet B; UVC, ultraviolet C; K_2_SO_4_, Potassium sulfate.

**Figure 9:**
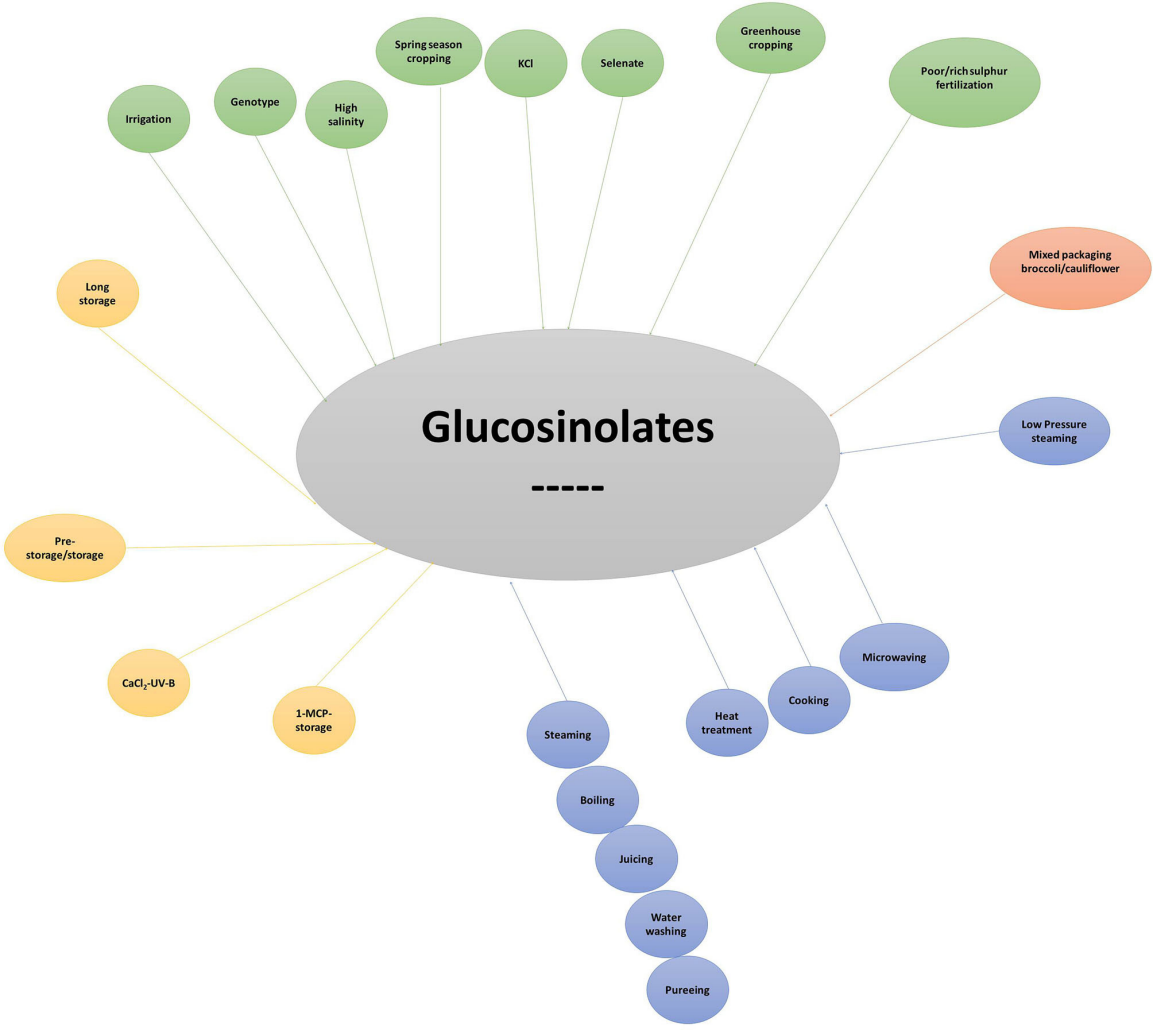
Major pre- and post-harvest factors negatively affecting glucosinolate content in broccoli. KCl, Potassium chloride; 1-MCP, 1-Methylcyclopropene, UV-B, ultraviolet B; CaCl_2_, Calcium Chloride.

GLs and their hydrolytic products isothiocyanates are increasingly attracting interest due to their various health benefits since their consumption was correlated with lower risks of various chronic degenerative diseases. As demonstrated, the levels of total or individual GLs from different classes may possibly fluctuate differently when exposed to various pre- (genotypes, growing environment, cultural practices, ripening stage, etc.) and post-harvest manipulations and agricultural procedures (harvesting, post-harvest treatments, packaging, storage, etc.) (Figures 8, 9). The understanding of these traits is crucial to improve, preserve, and maintain the nutritional quality of broccoli florets. These data are critical for breeders, processors, and producers willing to (1) improve the levels of GLs in broccoli genotypes via new breeding programs integrating large number of broccoli lines and good agricultural practices, such as salinity, water stress, and various elicitors; (2) adopt post-harvest procedures maintaining fresh-like quality and significant gain in GLs, such as refrigerated storage and UV treatments; and (3) adopt processing techniques and treatments leading to minimal loss of these highly bioactive compounds in broccoli genotype, such as pulsed light treatment, pulsed electric field, and UV radiation.

## Author Contributions

RI conceptualized the idea of this review. RI, IT, ZP, MS, ML, AM, TR'H, CH, HL, and FH scanned the literature, retrieved and processed papers referenced in the review, and wrote the manuscript. All authors critically reviewed the manuscript, enriched, developed the main parts, and contributed during the preparation of the tables and figures before submission.

## Conflict of Interest

The authors declare that the research was conducted in the absence of any commercial or financial relationships that could be construed as a potential conflict of interest.
